# Supplementary figures and images for: Influenza A infection accelerates disease-associated microglia formation during physiological aging
Source: bioRxiv. 2025 Dec 14:2025.12.11.693336. Preprint. [Version 1] doi: 10.64898/2025.12.11.693336 (PMC12710644; doi:10.64898/2025.12.11.693336)

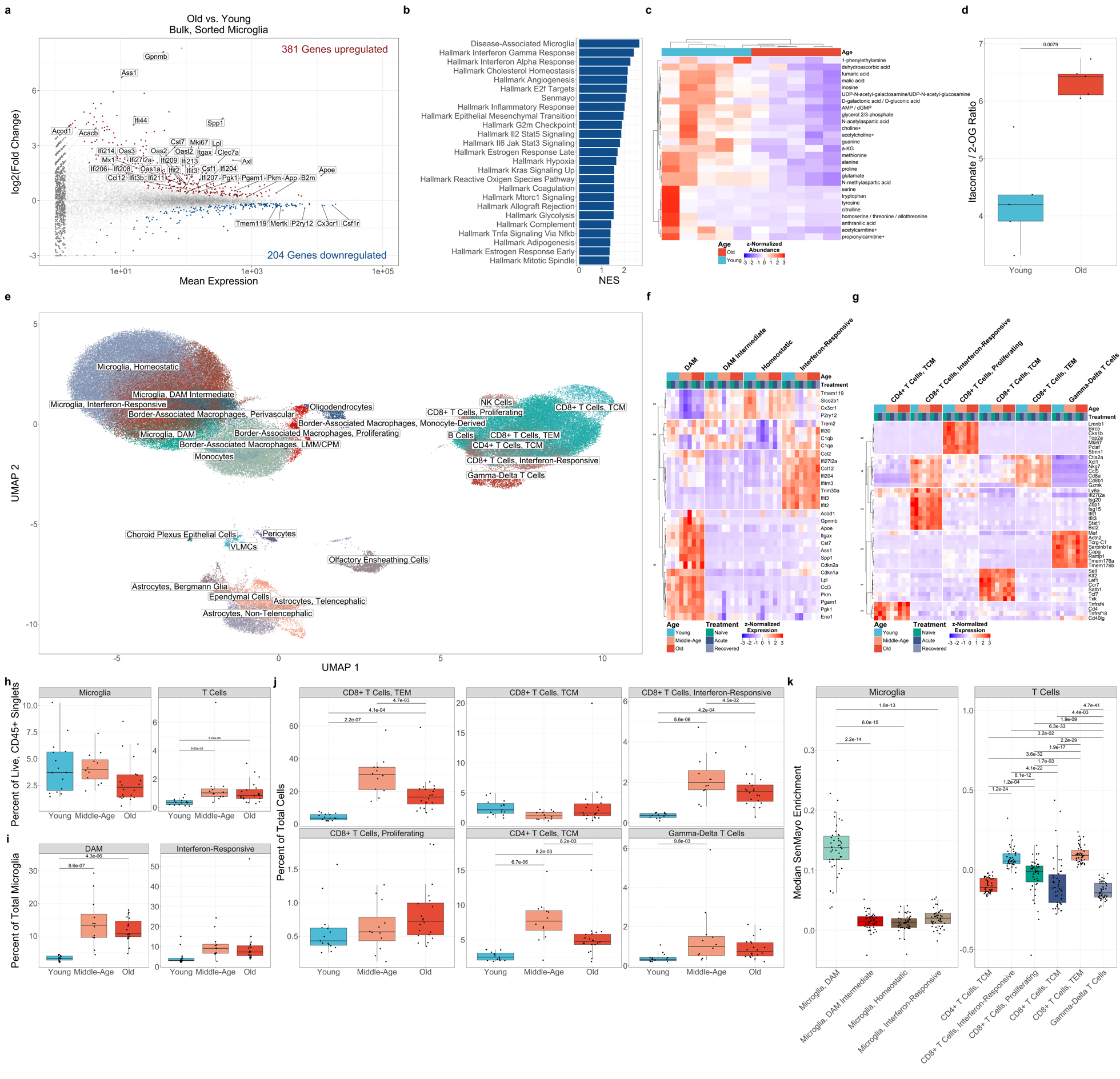

Supplement: Supplement 1 [file media-1.pdf]

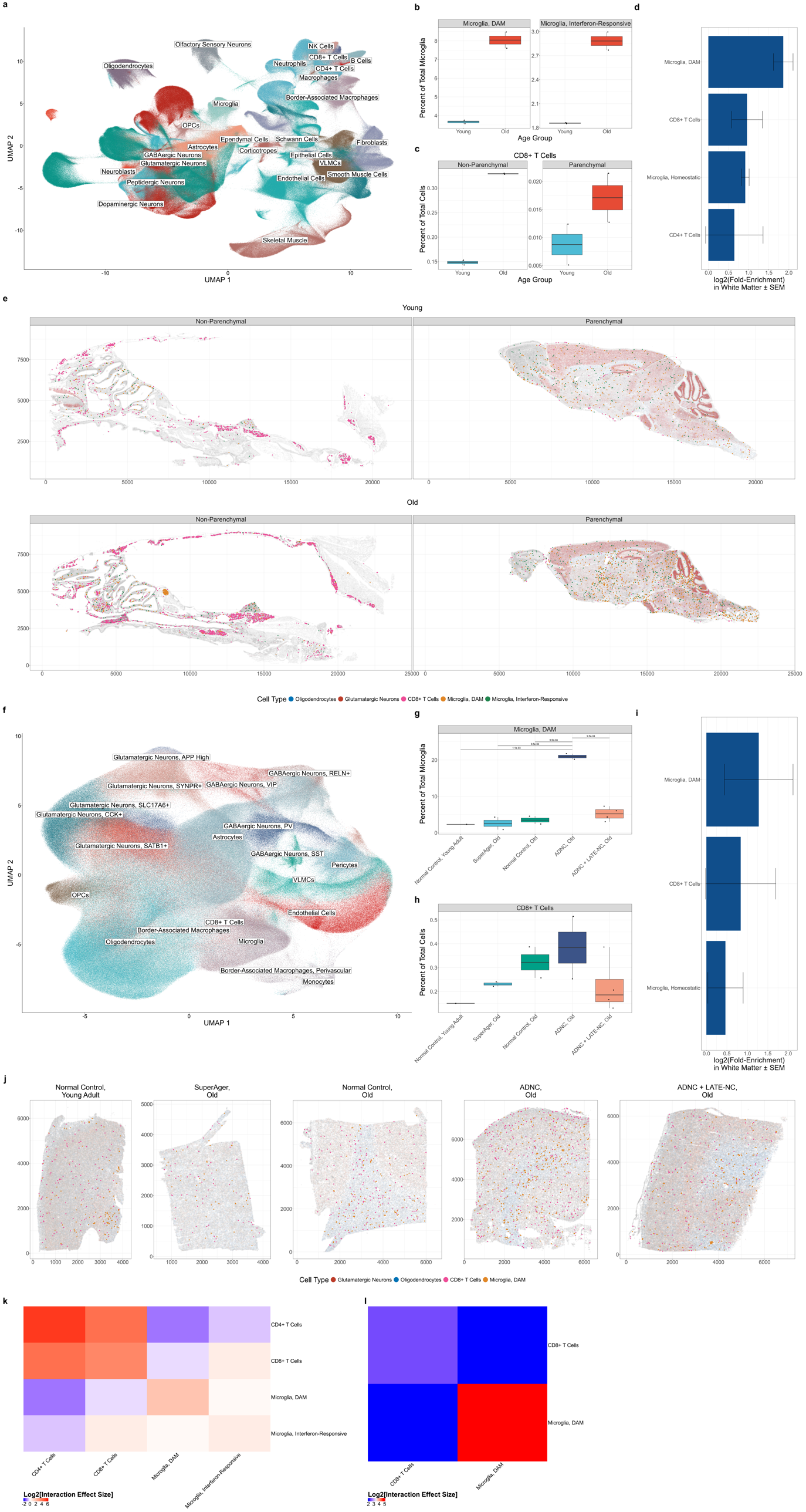

Supplement: Supplement 2 [file media-2.pdf]

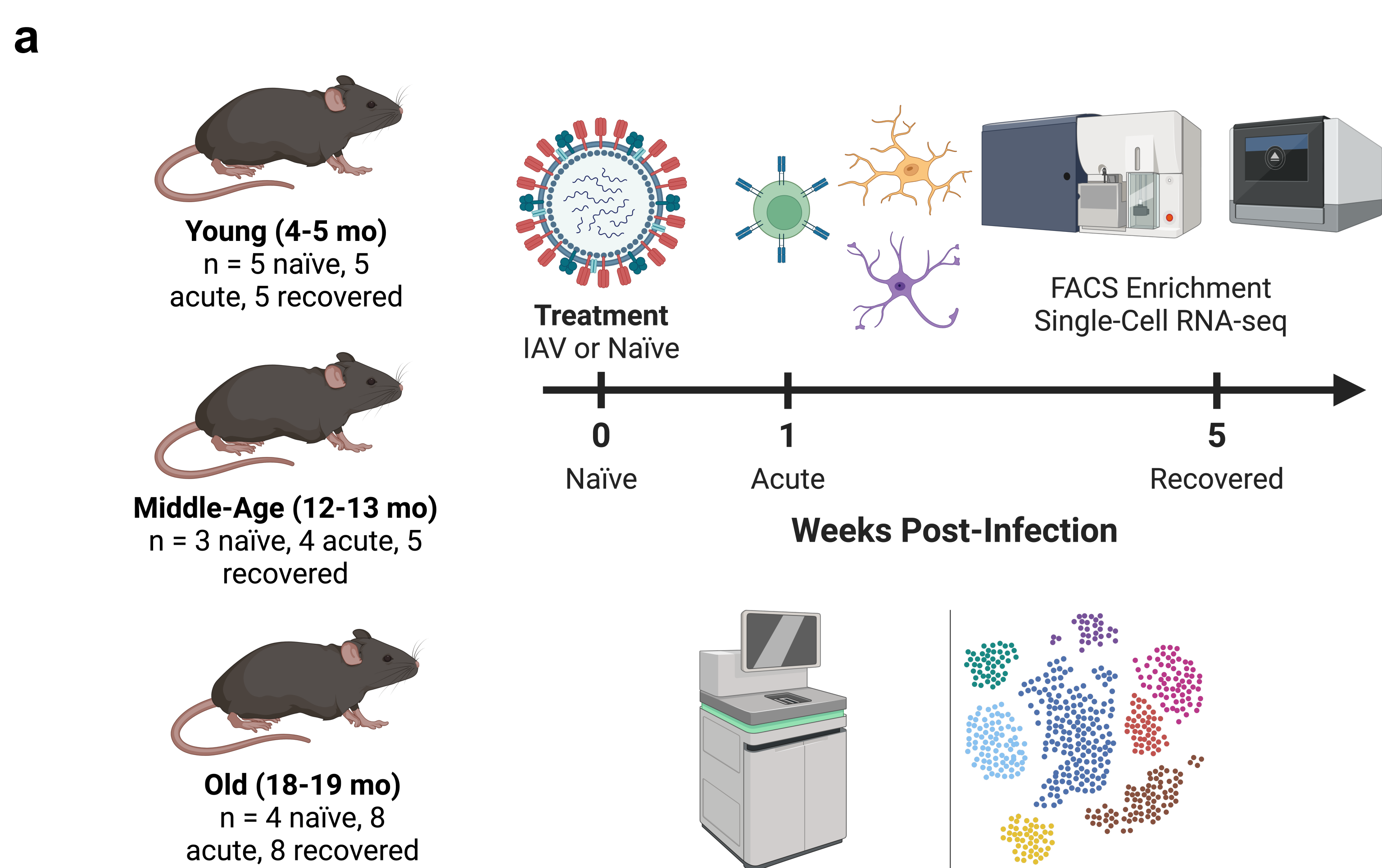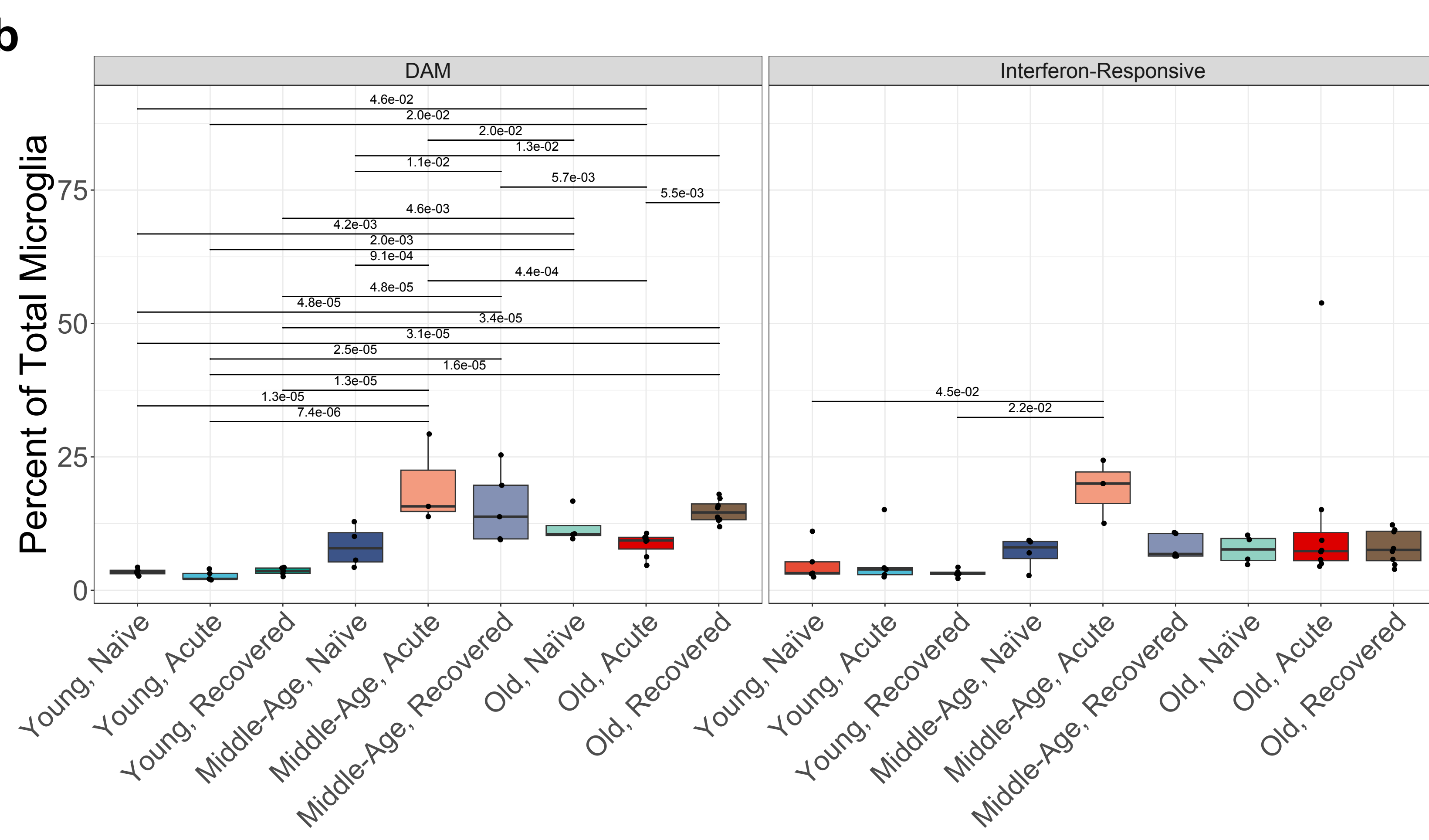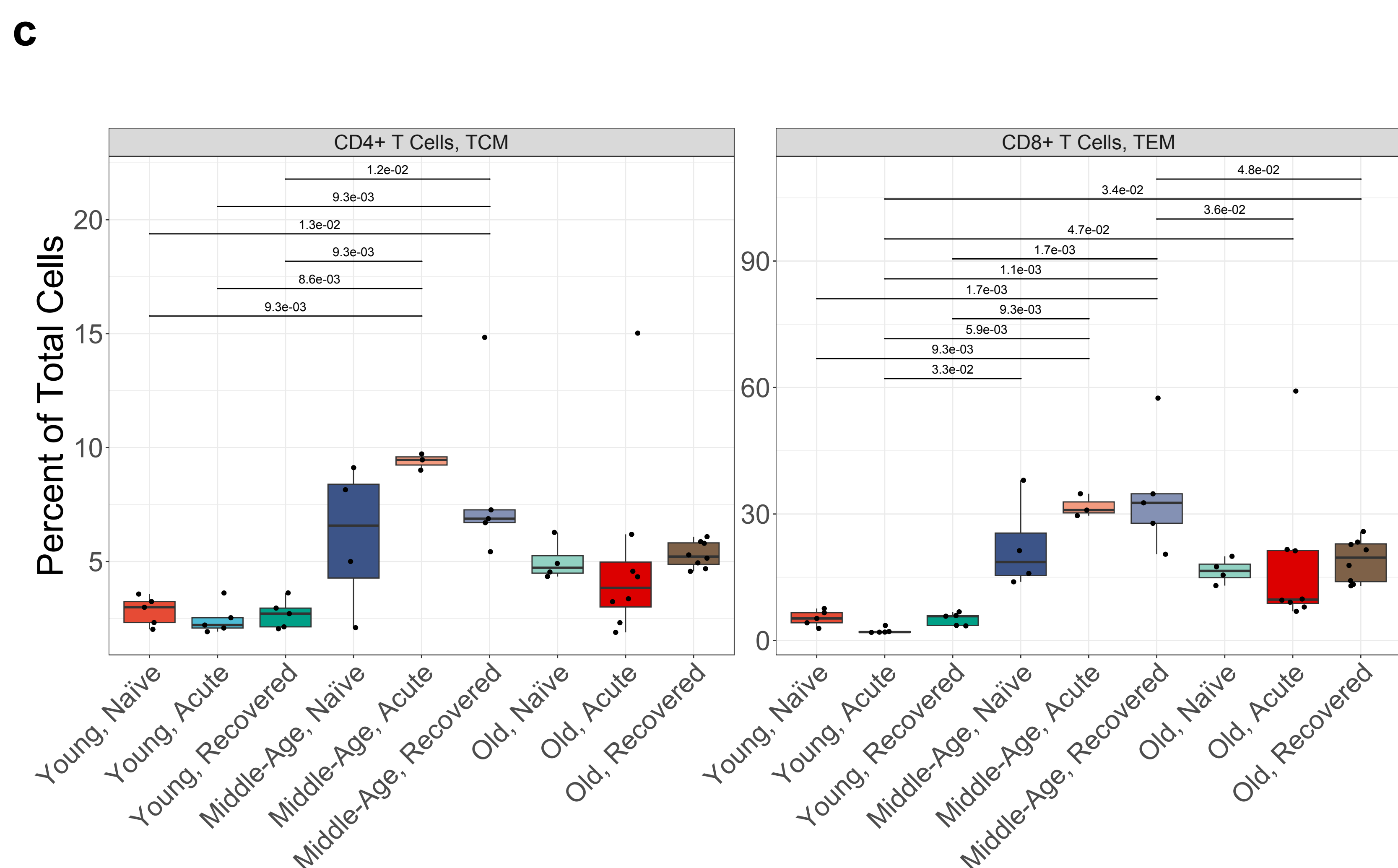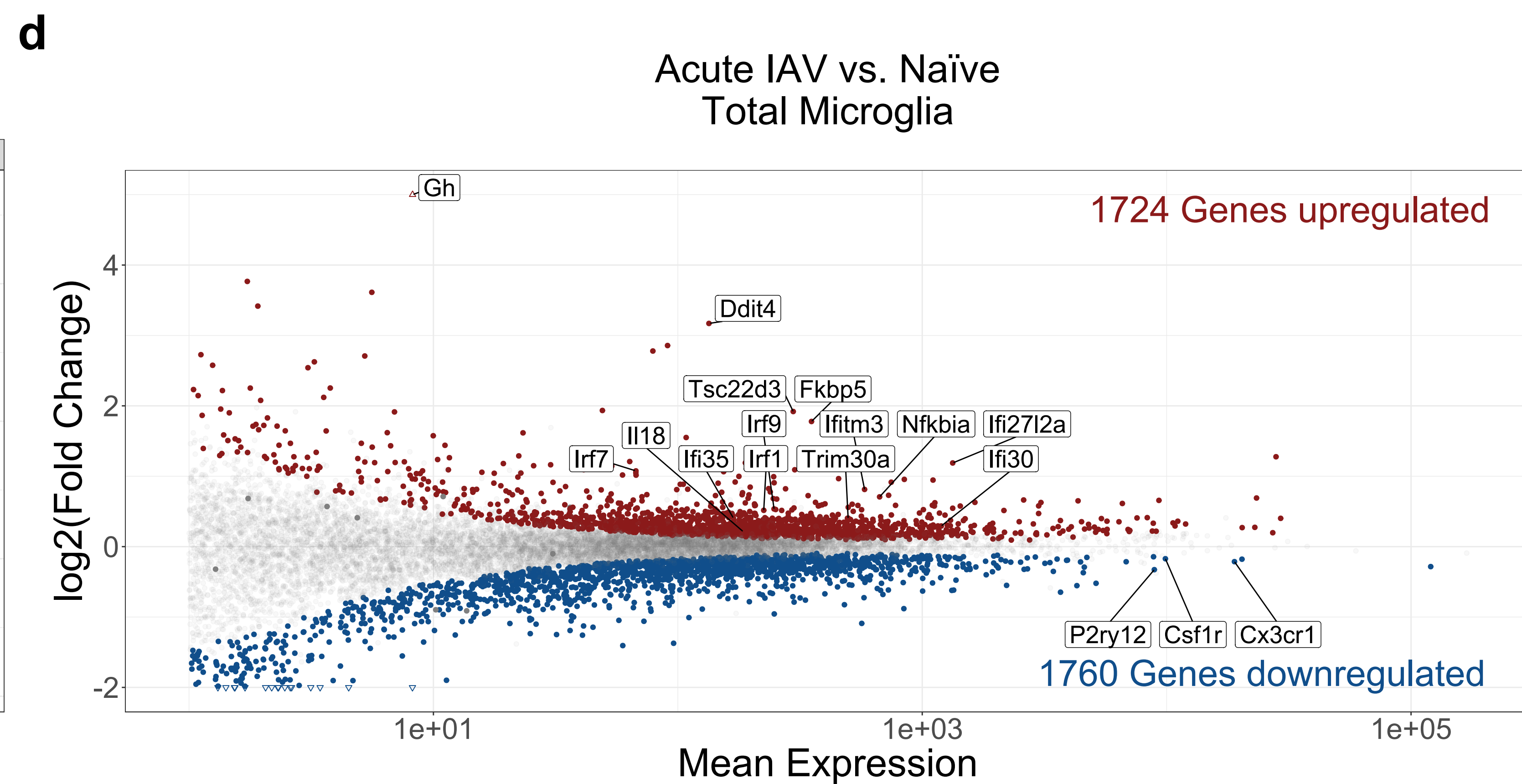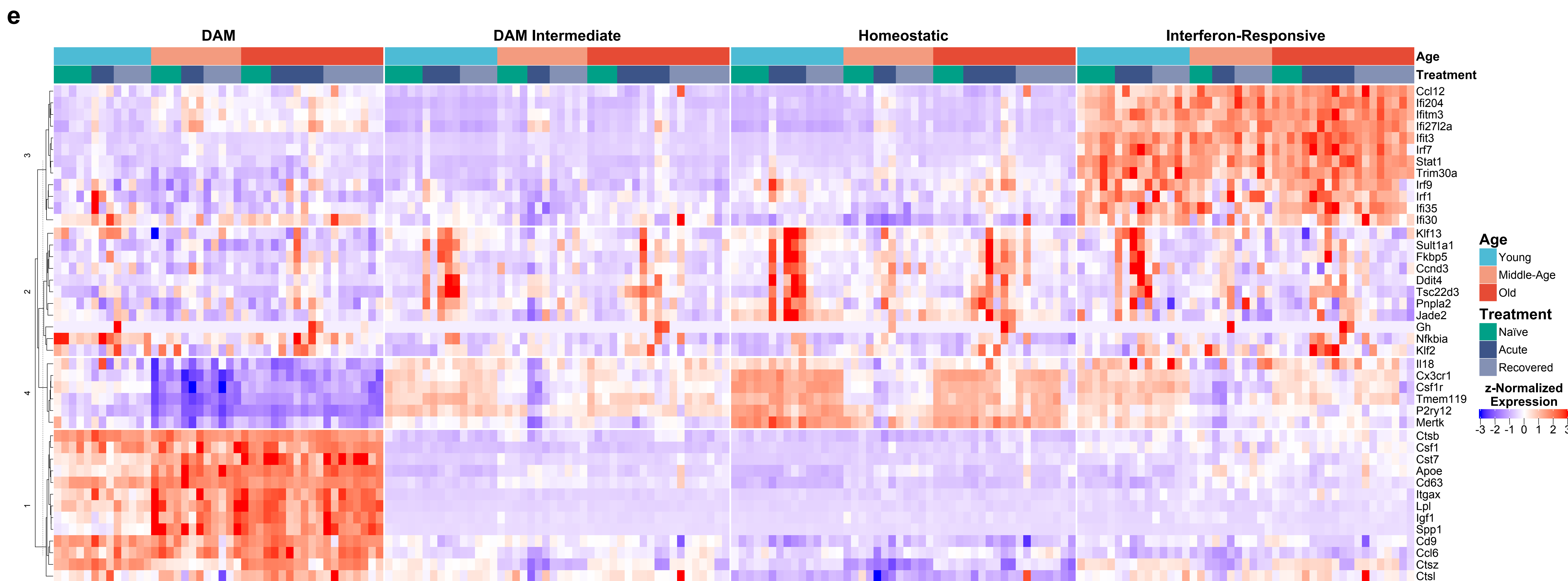

Supplement: Supplement 3 [file media-3.pdf]

**a**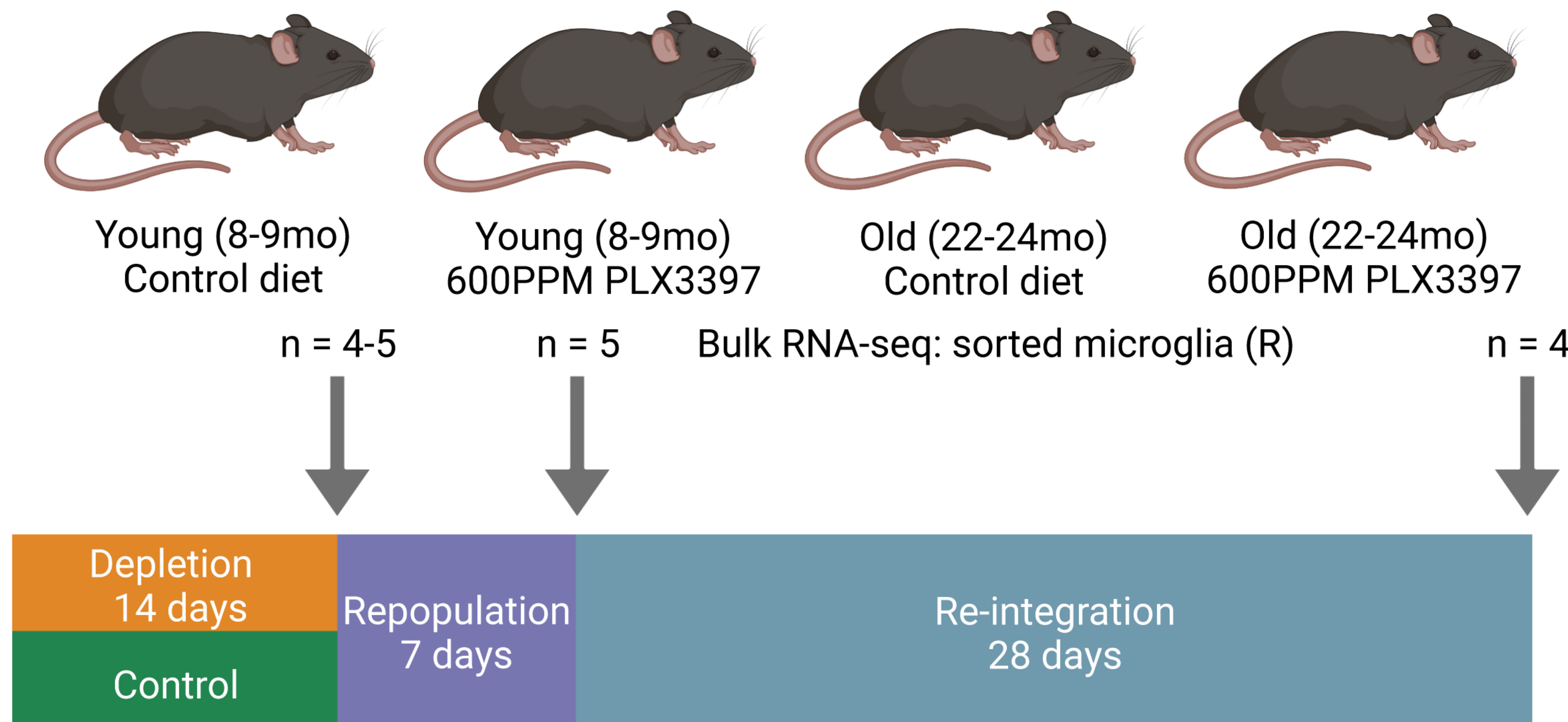**b**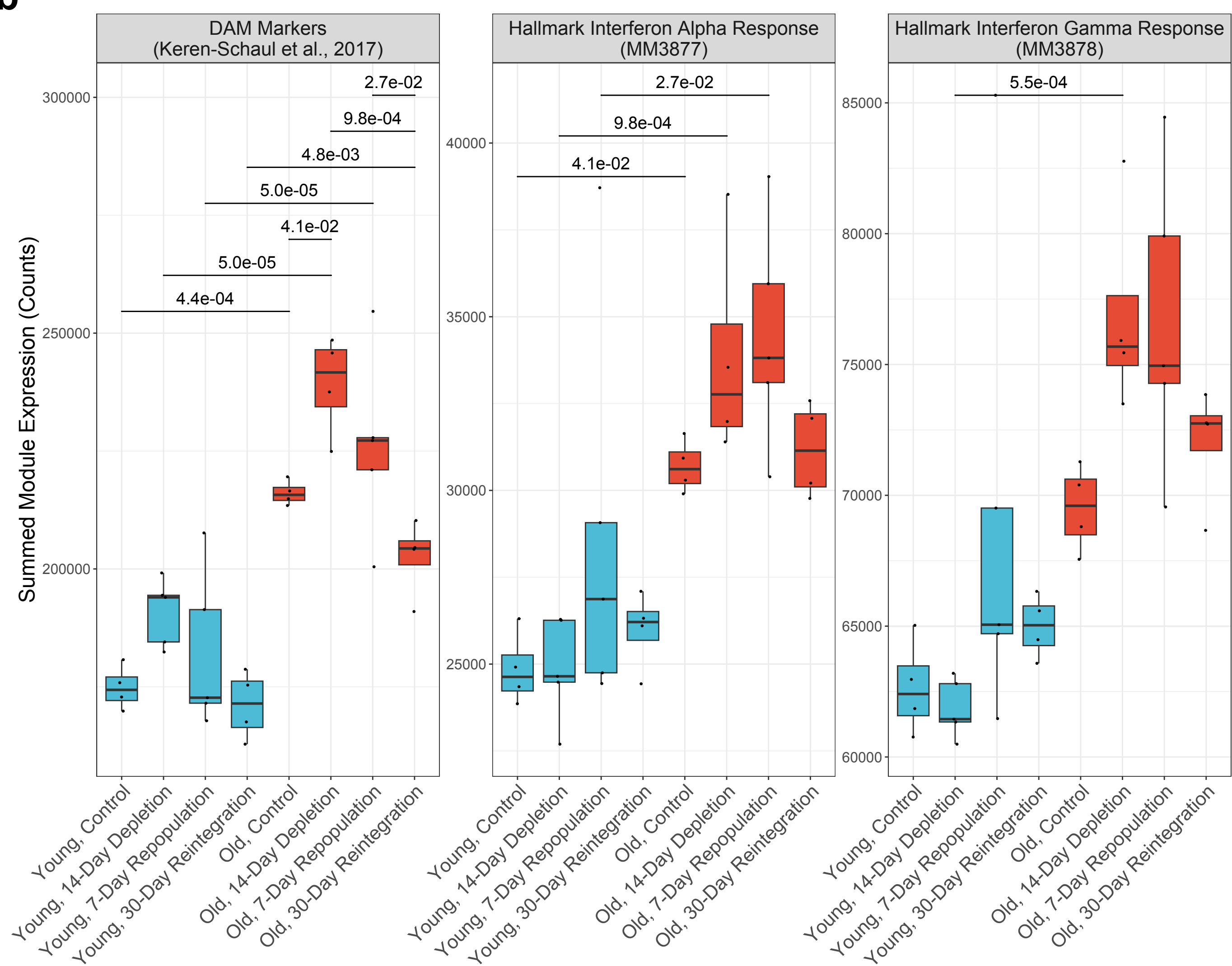**c**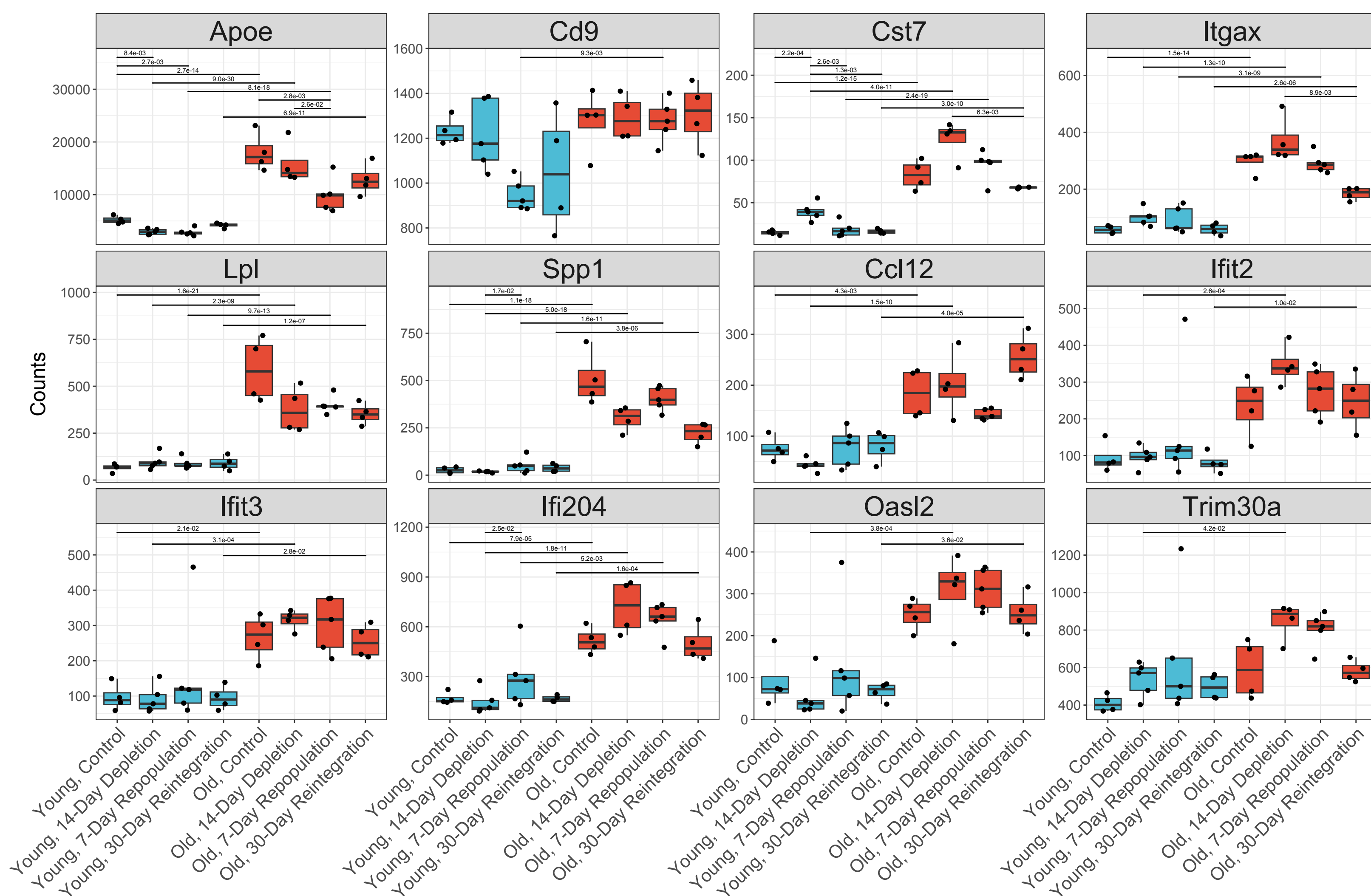**d**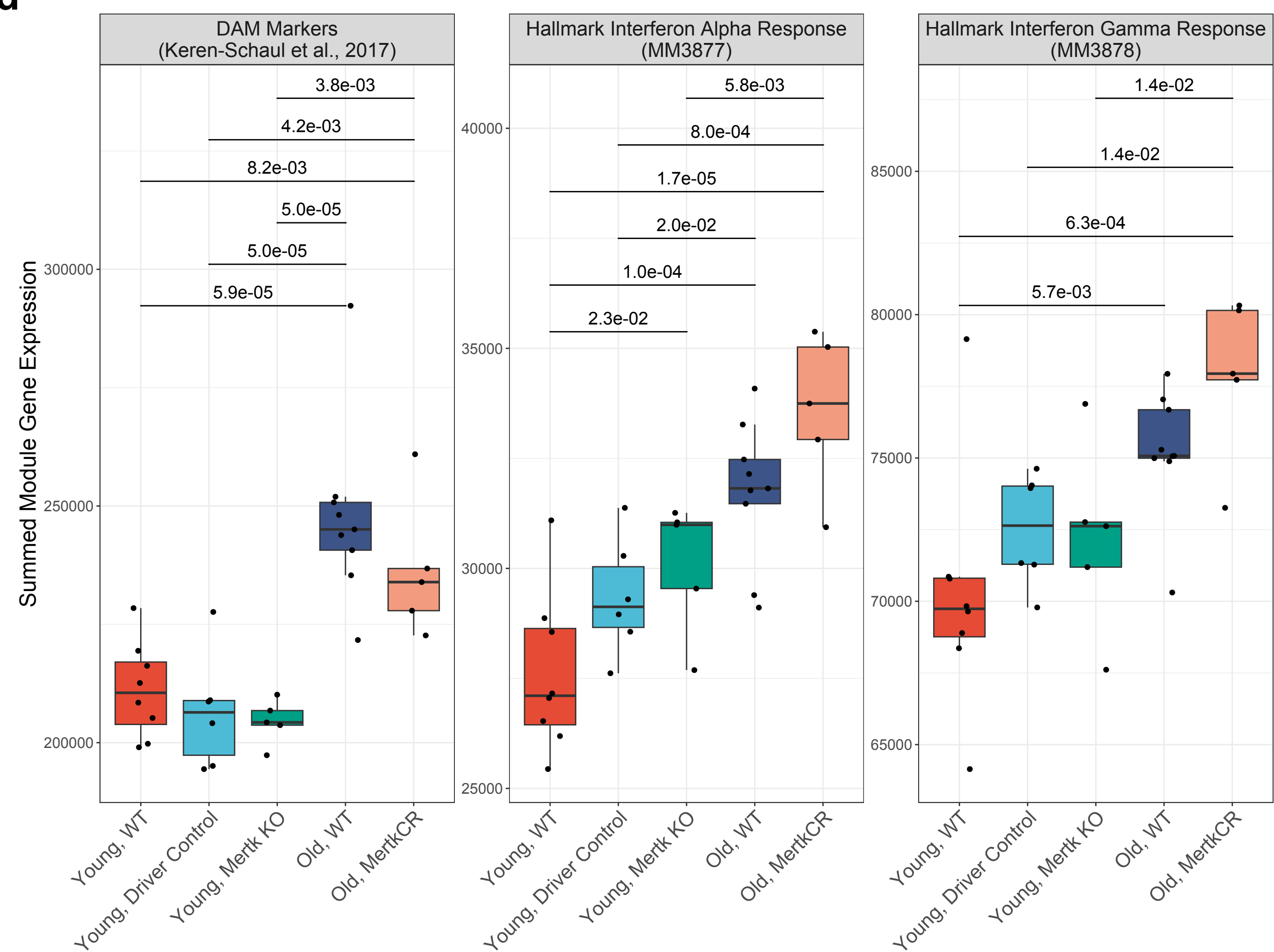**e**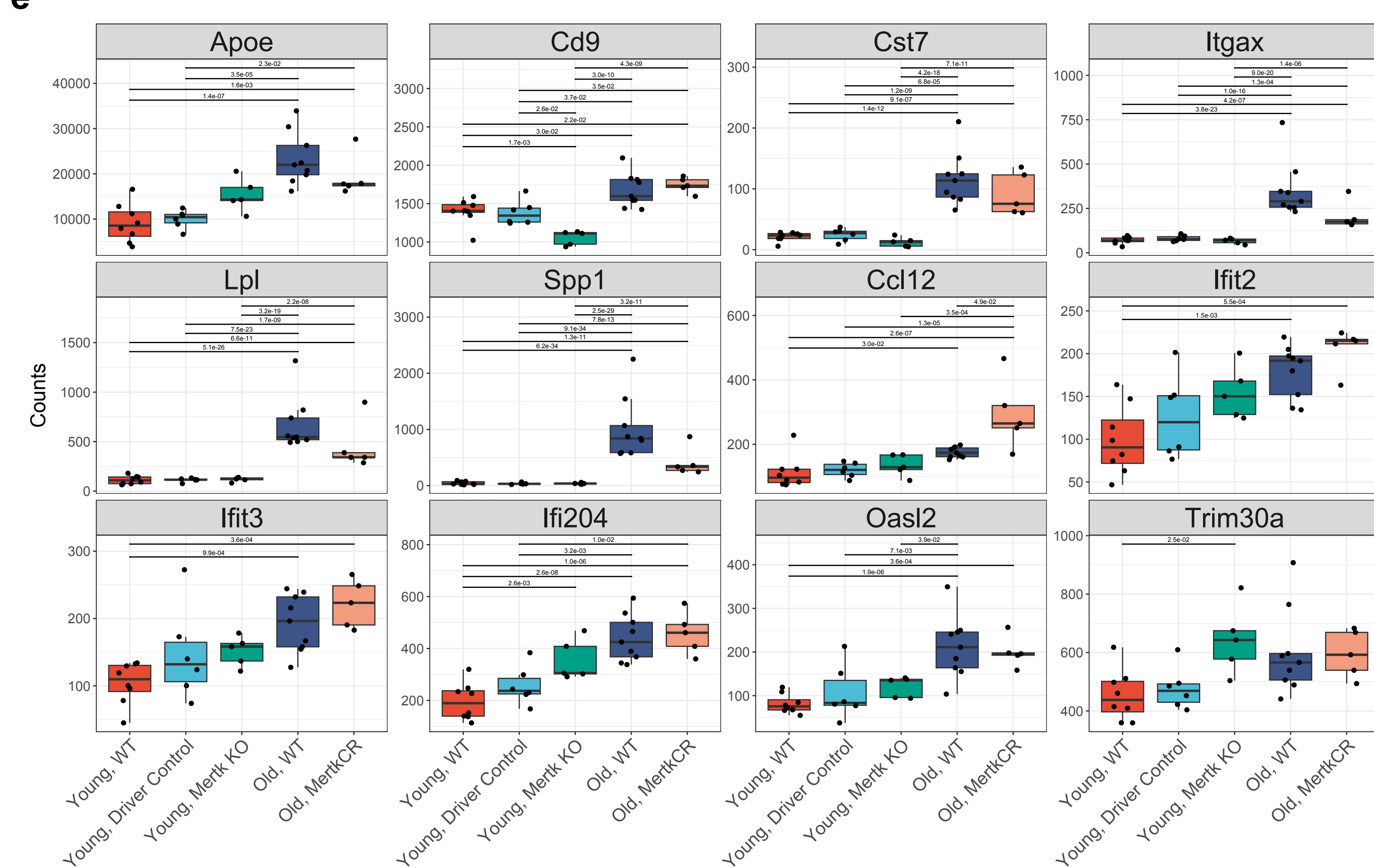

Supplement: Supplement 4 [file media-4.pdf]

**a**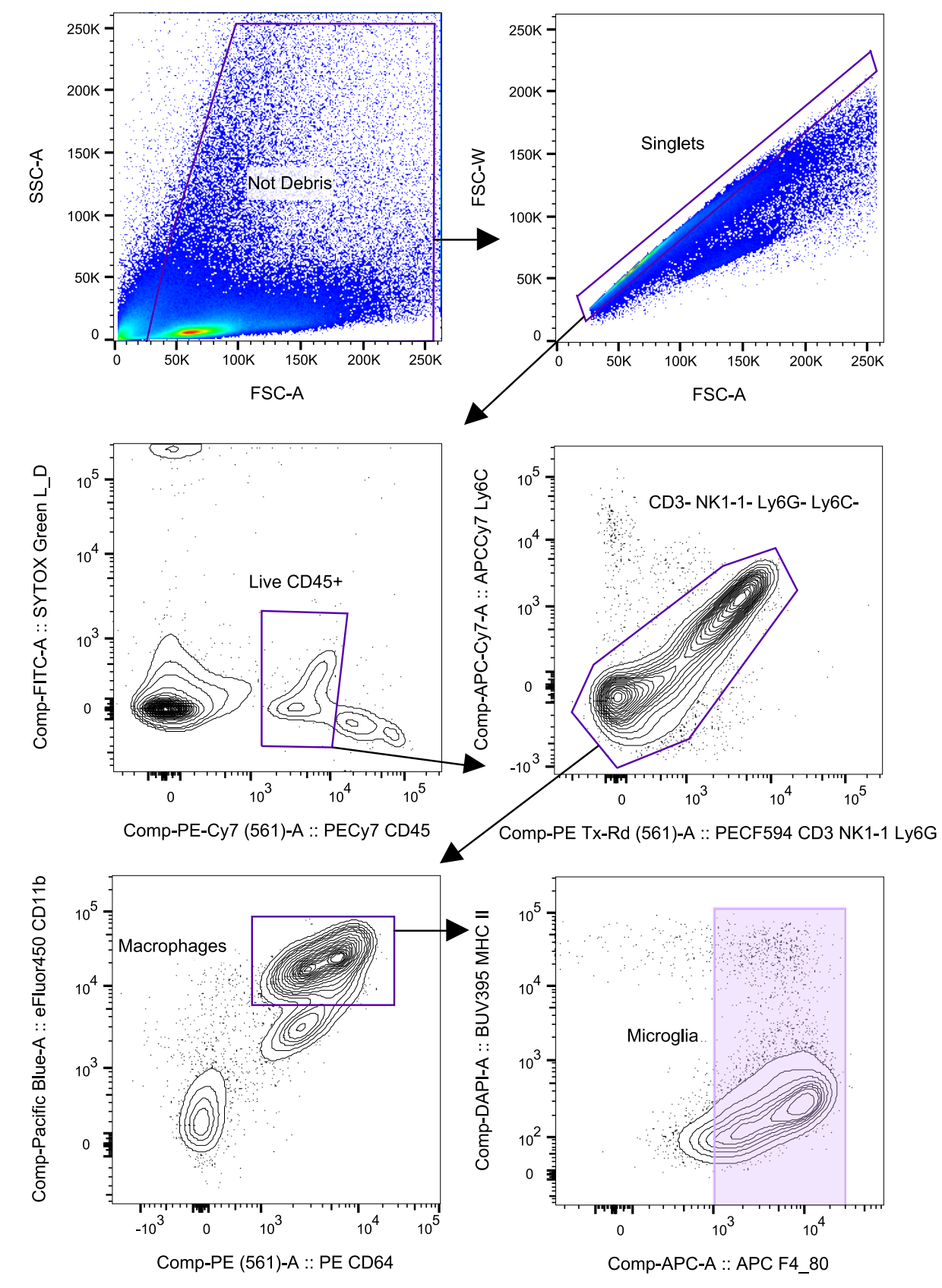**b**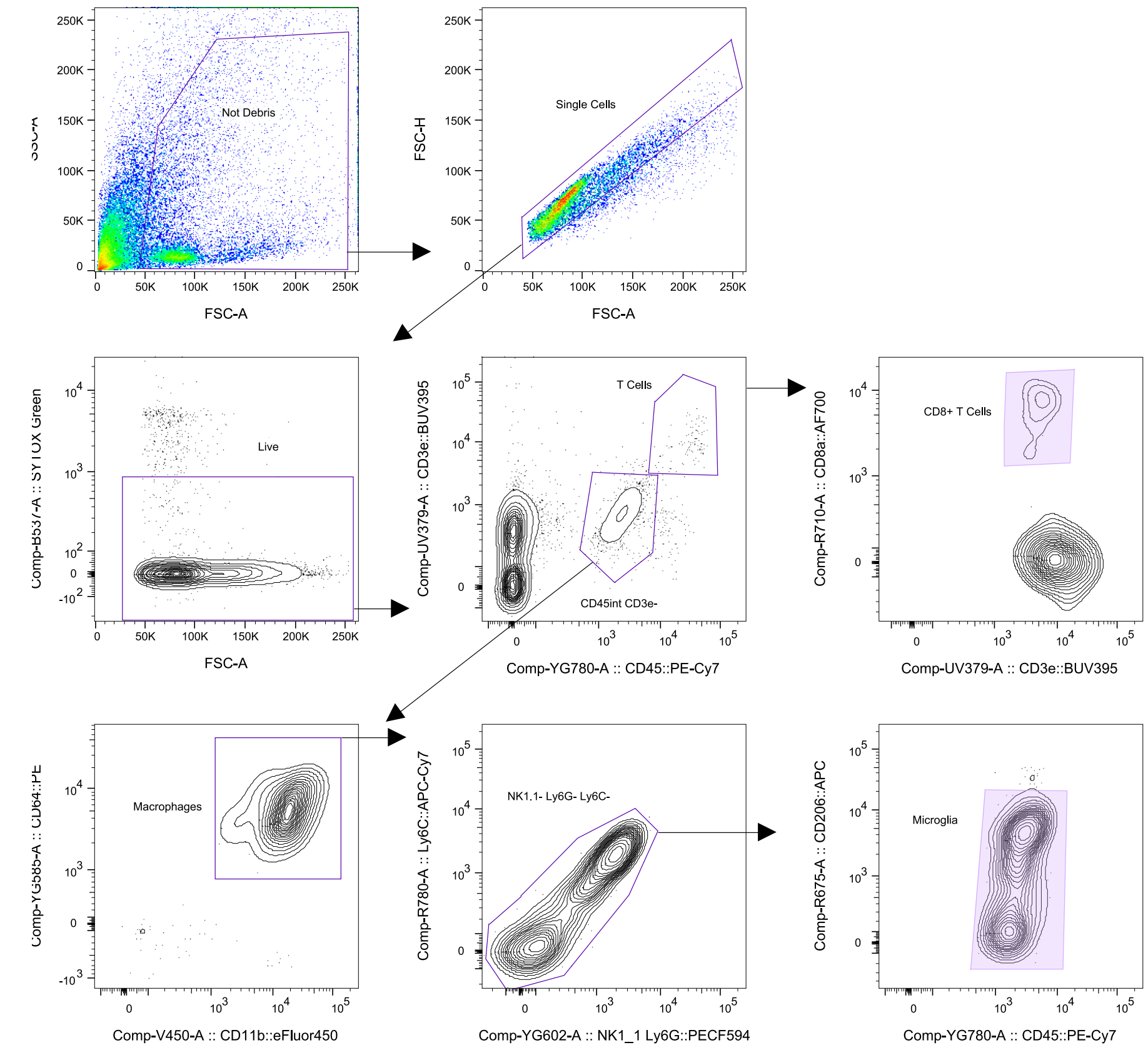**c**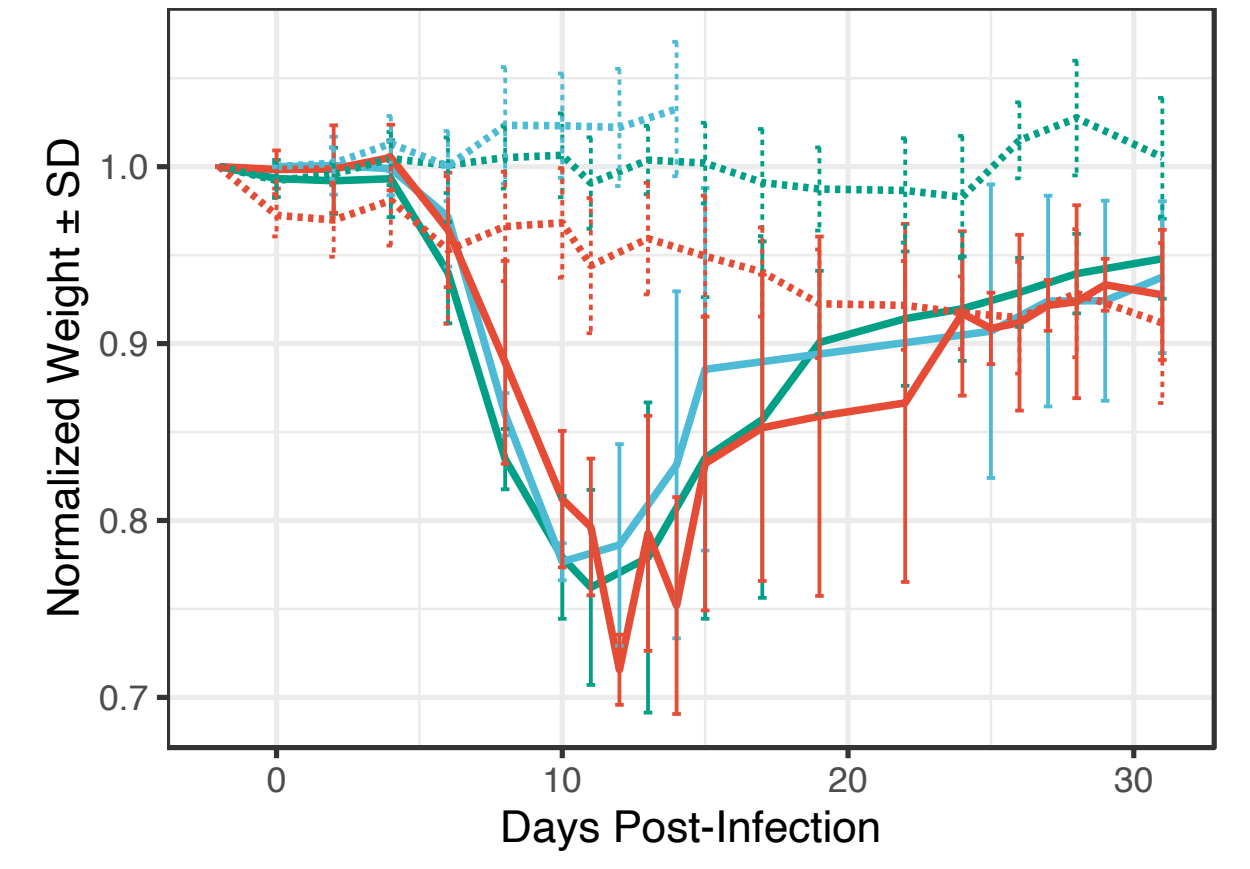**d**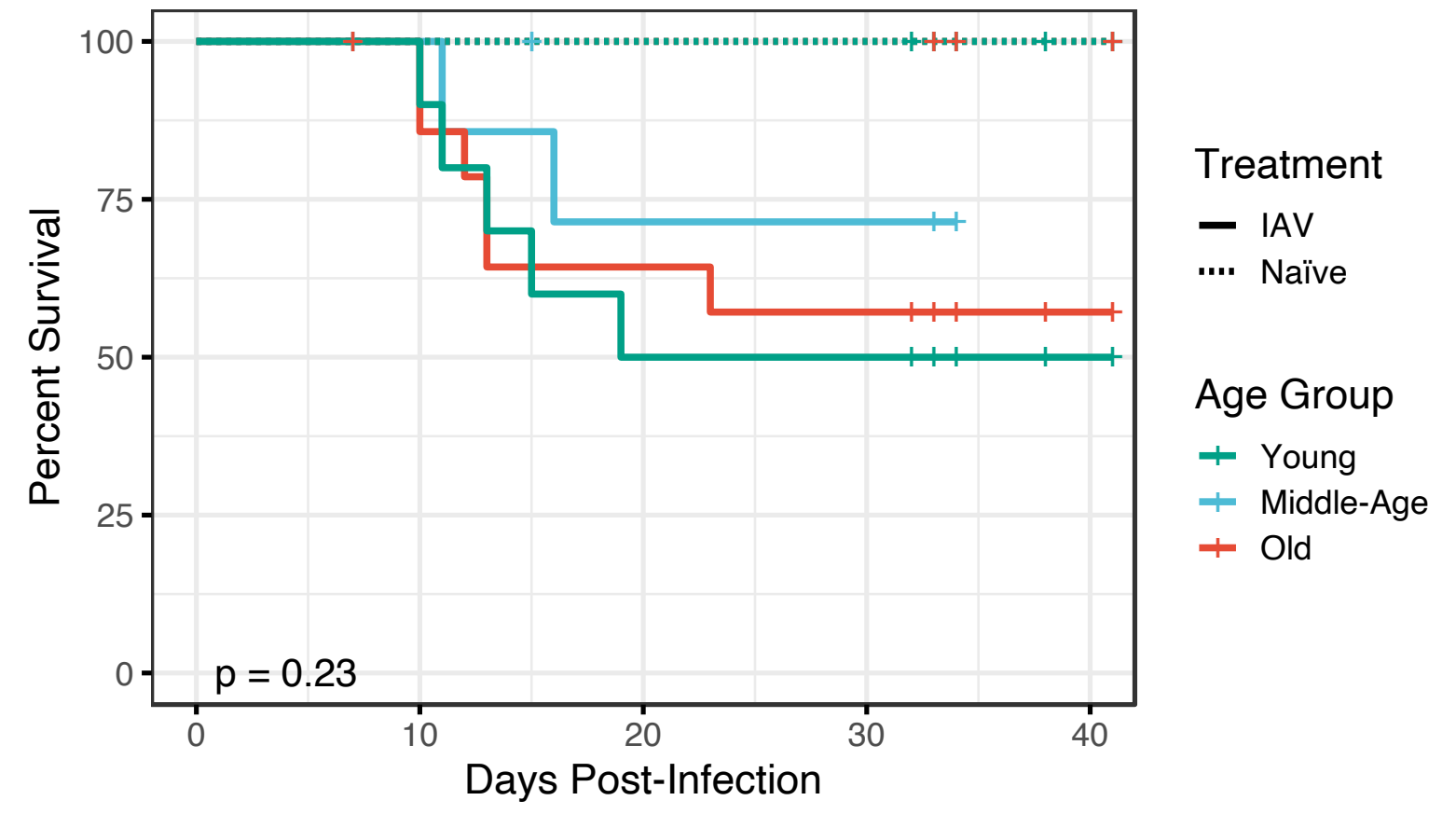**e**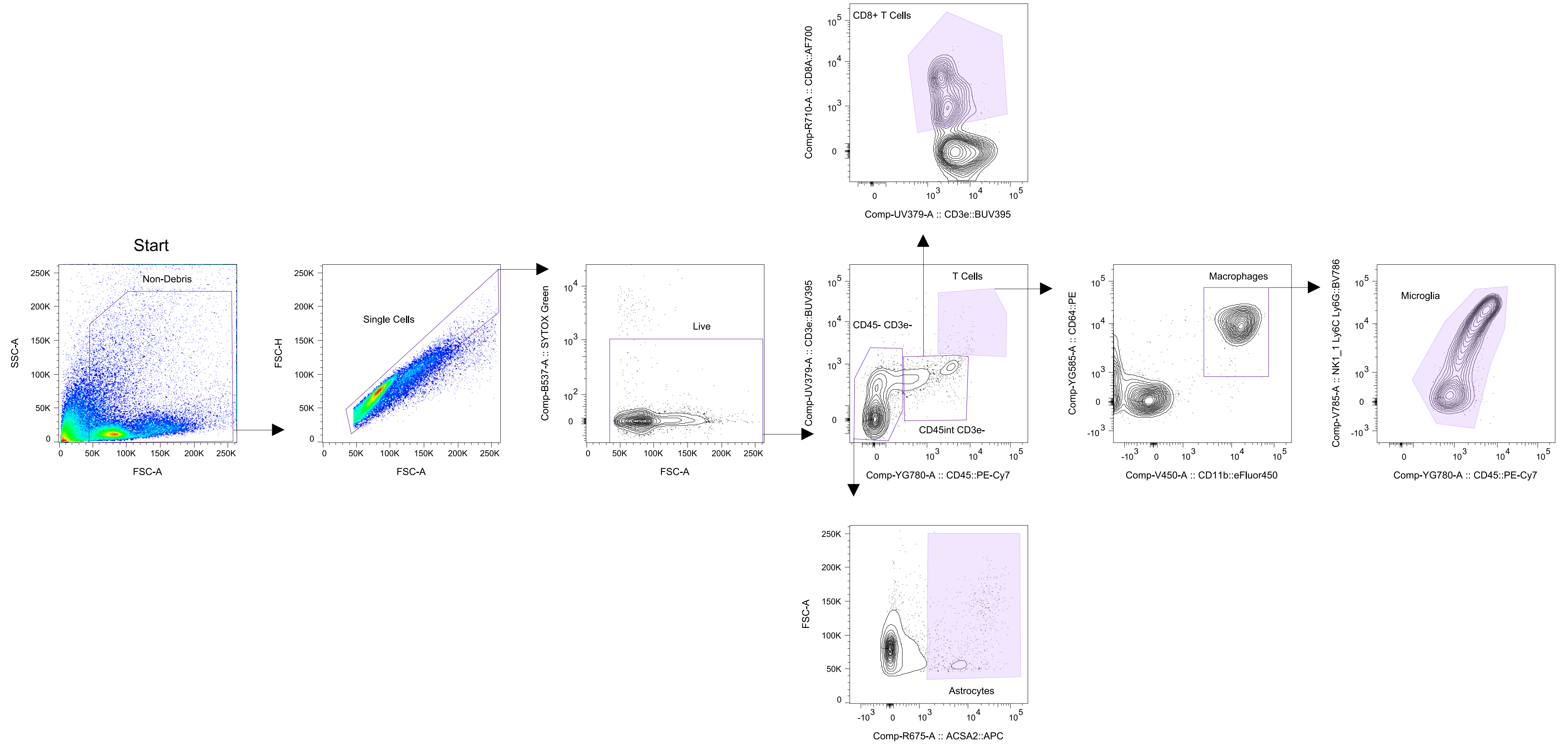

Supplement: Supplement 5 [file media-5.pdf]

a

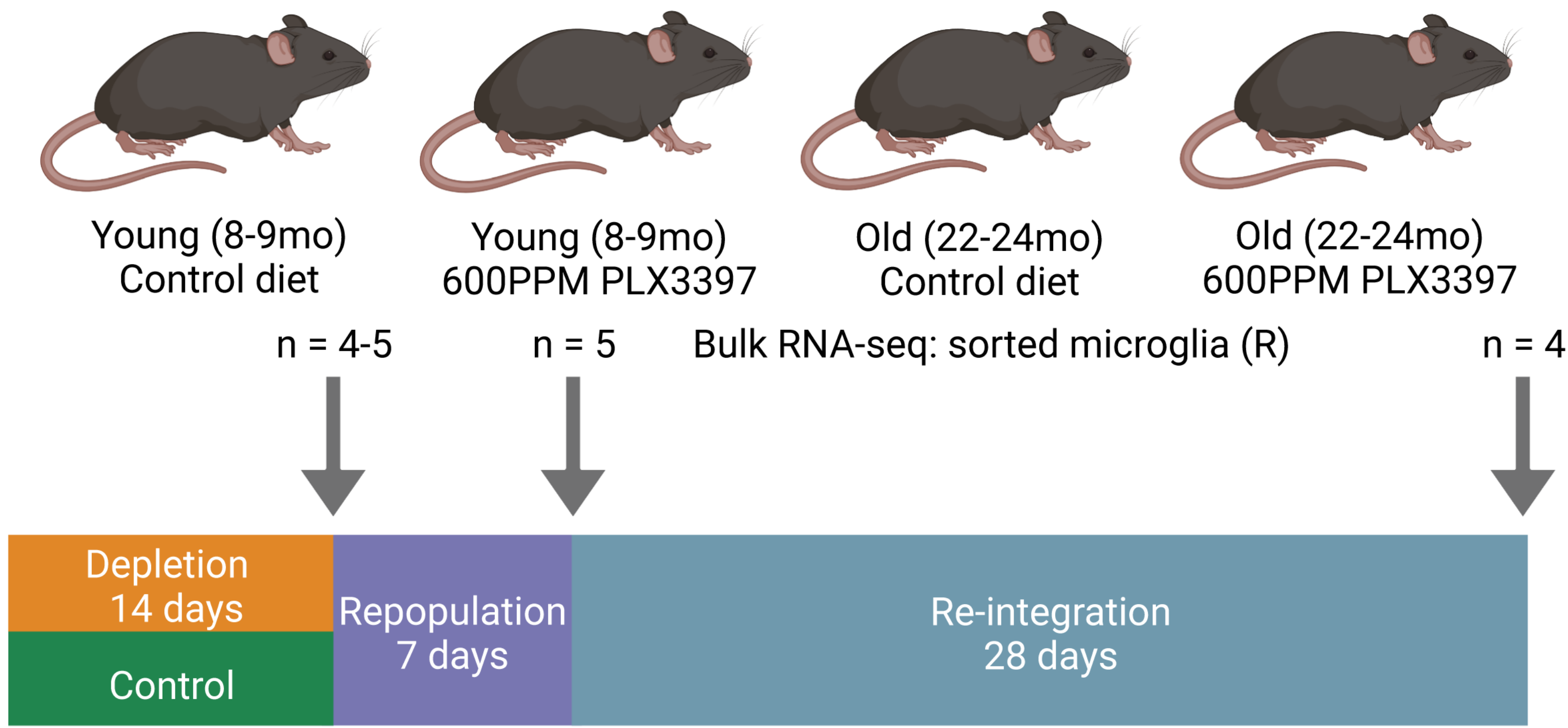

b

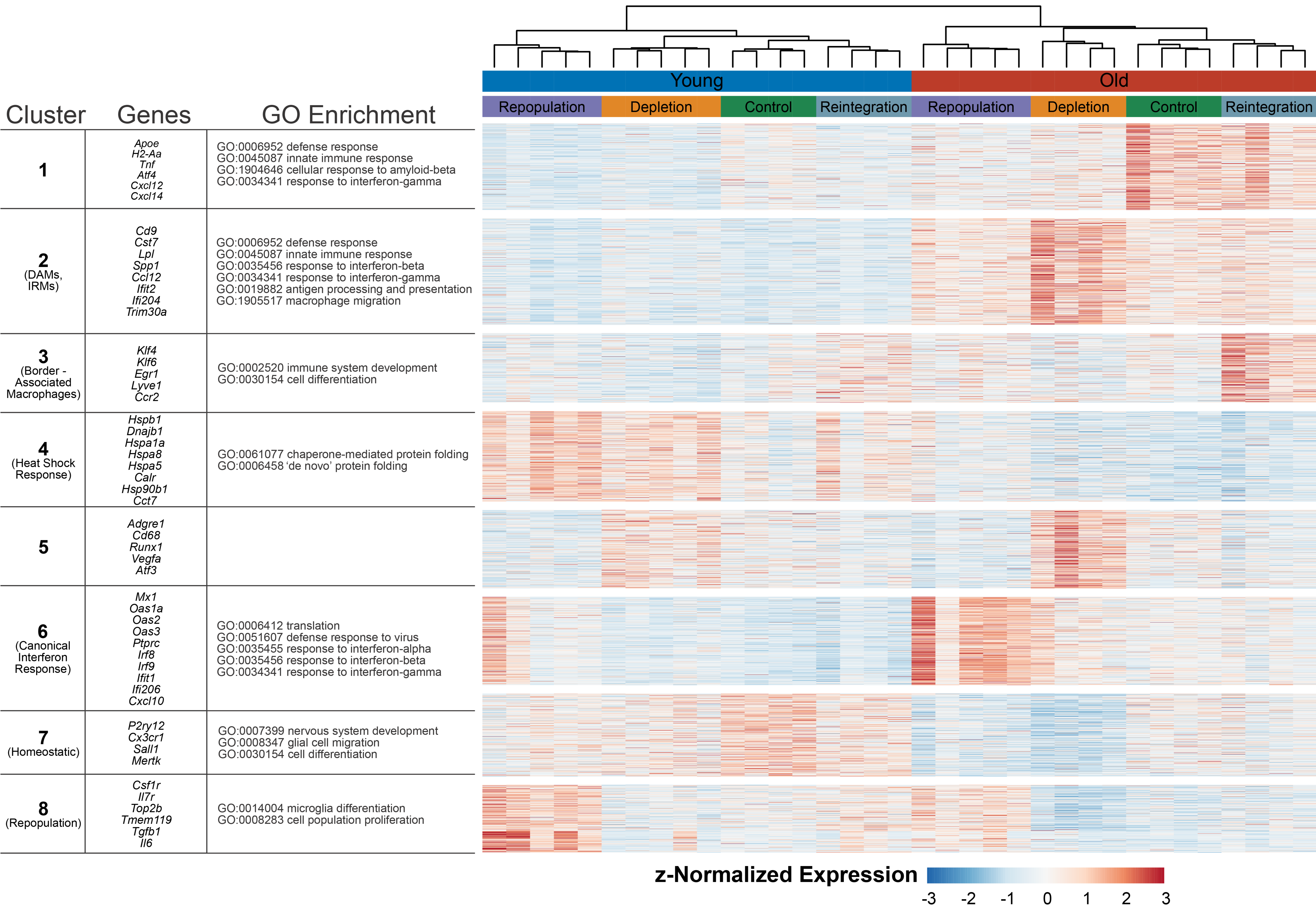

c

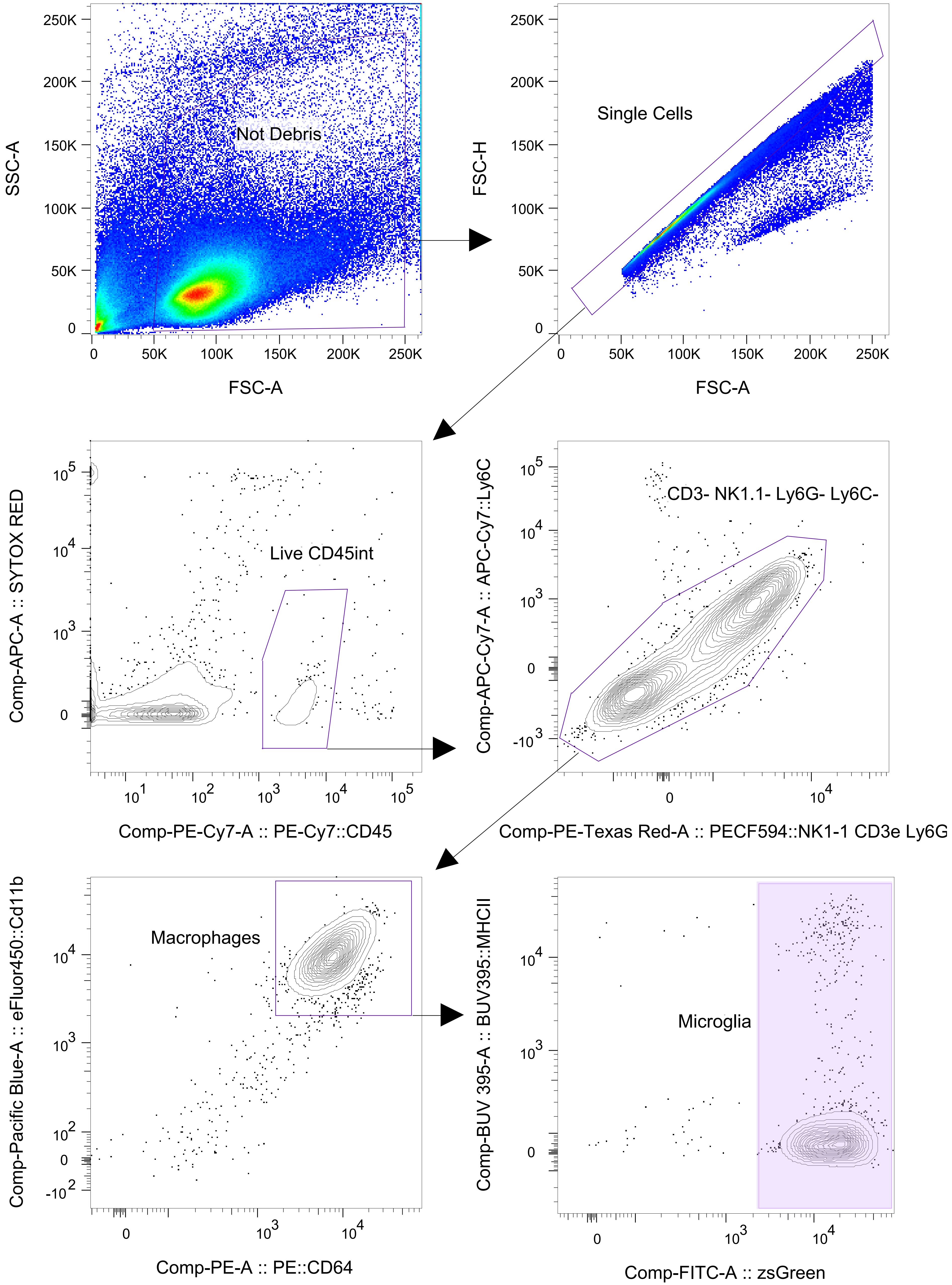

Supplement: Supplement 8 [file media-8.pdf]
