# Supplementary material for: Influenza A infection accelerates disease-associated microglia formation during physiological aging
Source: bioRxiv. 2025 Dec 14:2025.12.11.693336. Preprint. [Version 1] doi: 10.64898/2025.12.11.693336 (PMC12710644; doi:10.64898/2025.12.11.693336)

a

IAV Gene	Total Counts in Dataset	Mean Counts per Cell	SD
HA	0	0	0
M1	0	0	0
M2	0	0	0
NA	0	0	0
NEP	0	0	0
NP	0	0	0
NS1	0	0	0
PA	0	0	0
PA-X	0	0	0
PB1	0	0	0
PB1-F2	0	0	0
PB2	0	0	0

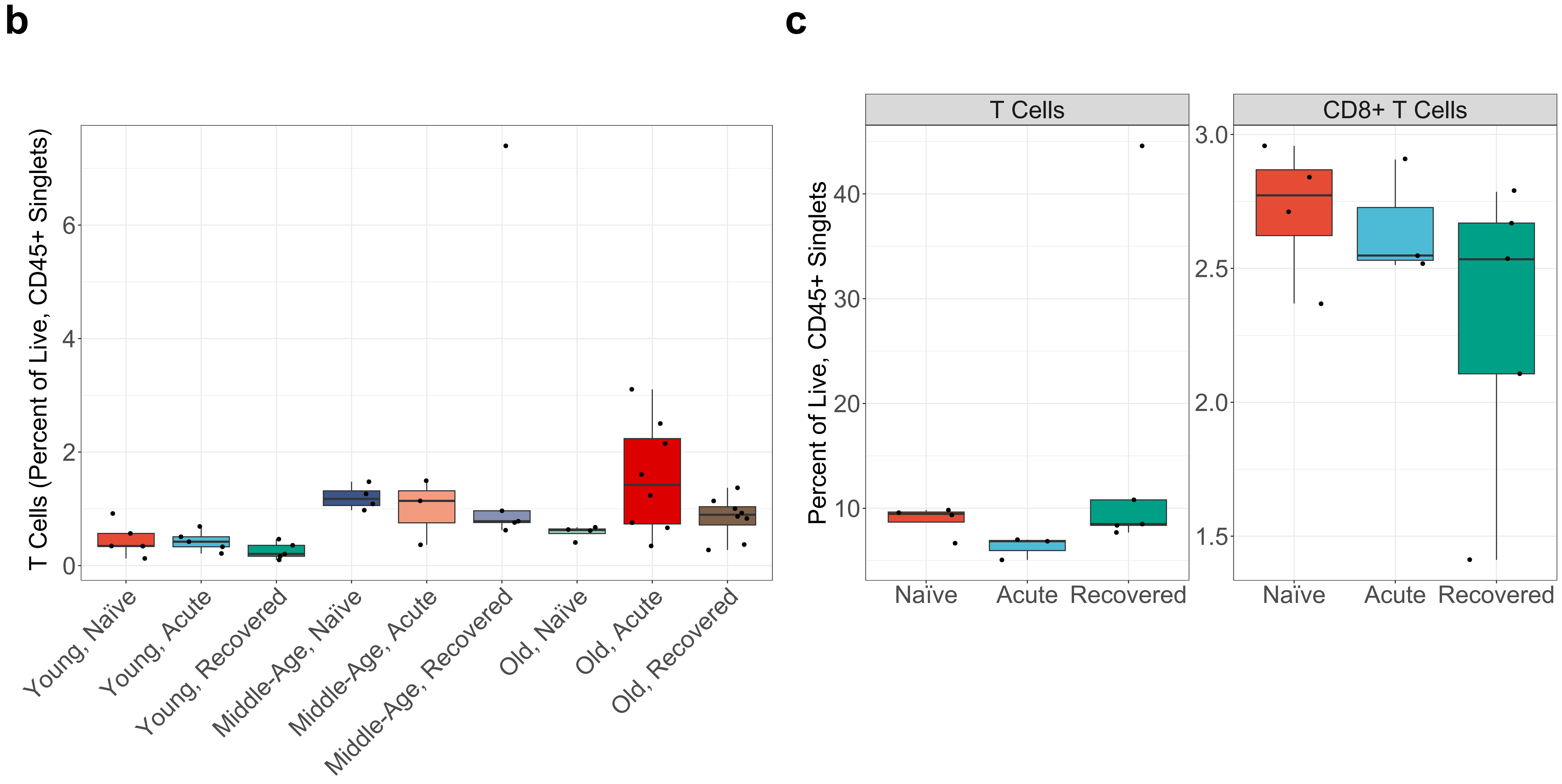

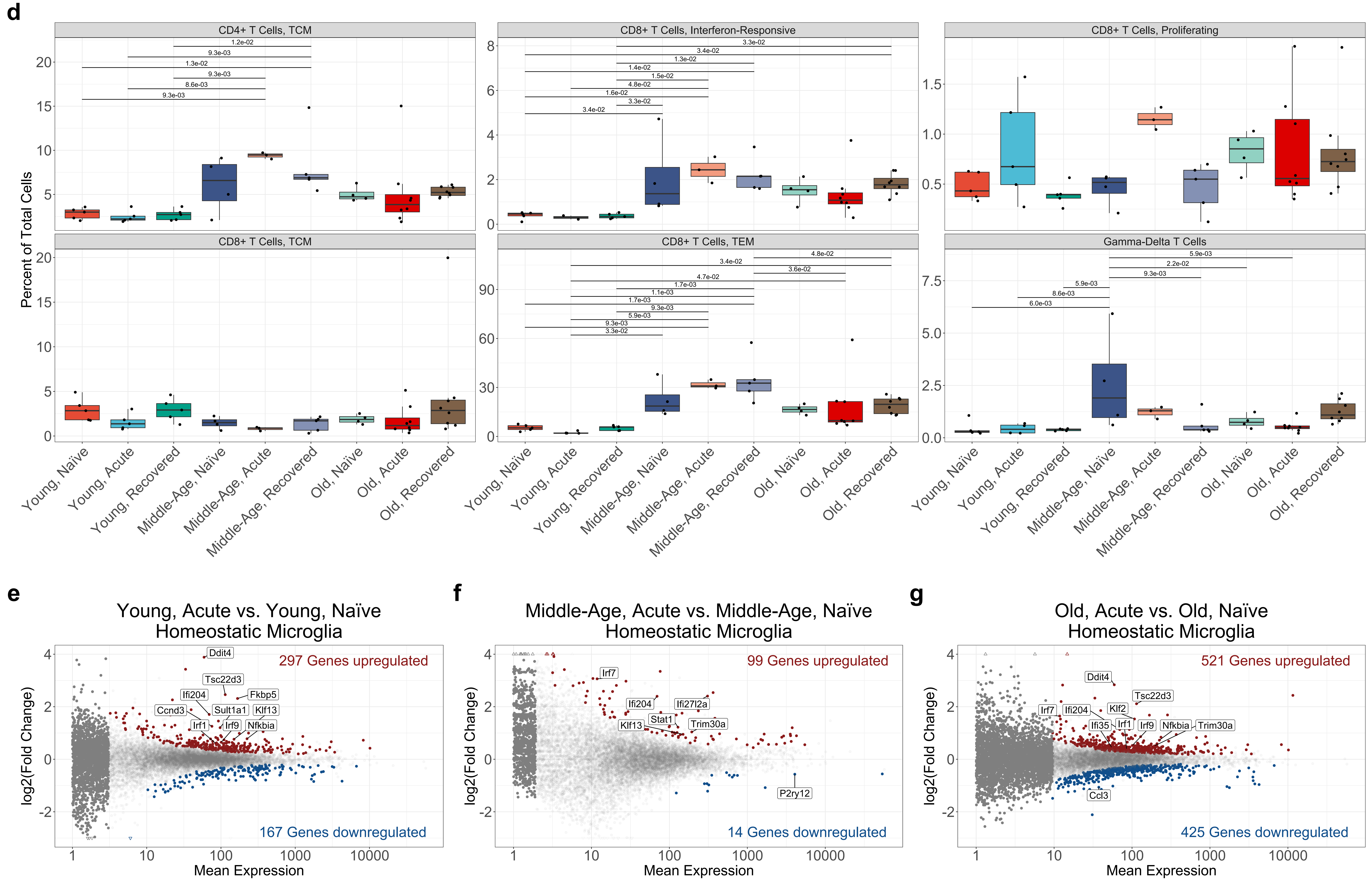

Supplement: Supplement 7 [file media-7.pdf]
