# Supplementary material for: Influenza A infection accelerates disease-associated microglia formation during physiological aging
Source: bioRxiv. 2025 Dec 14:2025.12.11.693336. Preprint. [Version 1] doi: 10.64898/2025.12.11.693336 (PMC12710644; doi:10.64898/2025.12.11.693336)
Supplement: Supplement 14 [file media-14.pdf]

Supplementary Table 4: Human brain aging cohort demographics

Case Number	Clinical Diagnosis	Primary Neuropathologic Diagnoses	NIA–AA ADNC (ABC Score)	ApoE	PMI (Hrs)	Sex	Age at Death	Race/Ethnicity	Group
1	Amnestic dementia	Intermediate ADNC, LATE stage 2	A3, B2, C2	3,3	8.5	Female	95	Caucasian/non-hispanic	ADNC + LATE-NC, Old
2	SuperAger	Intermediate ADNC, LATE stage 2	A3, B2, C3	3,3	20	Female	97	Caucasian/non-hispanic	ADNC + LATE-NC, Old
3	SuperAger	Low ADNC	A1, B1, C1	3,3	6	Male	91	Caucasian/non-hispanic	SuperAger, Old
4	Amnestic dementia	High ADNC, LATE stage 2	A3, B3, C3	3,4	11	Female	75	Caucasian/non-hispanic	ADNC + LATE-NC, Old
5	SuperAger	PART, LATE stage 2	A0, B2, C0	3,3	16	Male	87	Caucasian/non-hispanic	SuperAger, Old
6	SuperAger	PART	A0, B2, C0	3,3	9	Female	85	Caucasian/non-hispanic	SuperAger, Old
7	Young control	None	None	Unavailable	Unavailable	Male	28	Unavailable	Normal Control, Young Adult
8	Normal control	Intermediate ADNC, LATE stage 1	A3, B2, C1	3,4	21	Male	92	Caucasian/non-hispanic	Normal Control, Old
9	Amnestic dementia	High ADNC	A3, B3, C3	3,3	16	Male	62	Caucasian/non-hispanic	ADNC, Old
10	Amnestic dementia	High ADNC	A3, B3, C3	Unavailable	18	Male	66	Caucasian/non-hispanic	ADNC, Old
11	Normal control	Low ADNC	A3, B1, C3	3,3	16	Female	87	Caucasian/non-hispanic	Normal Control, Old
