# Supplementary material for: Influenza A infection accelerates disease-associated microglia formation during physiological aging
Source: bioRxiv. 2025 Dec 14:2025.12.11.693336. Preprint. [Version 1] doi: 10.64898/2025.12.11.693336 (PMC12710644; doi:10.64898/2025.12.11.693336)
Supplement: 1 [file NIHPP2025.12.11.693336V1-supplement-1.pdf]

bioRxiv preprint doi: https://doi.org/10.64898/2025.12.11.693336; this version posted December 14, 2025. The copyright holder for this preprint (which was not certified by peer review) is the author/funder, who has granted bioRxiv a license to display the preprint in perpetuity. It is made available under aCC-BY 4.0 International license.

Supplemental Data

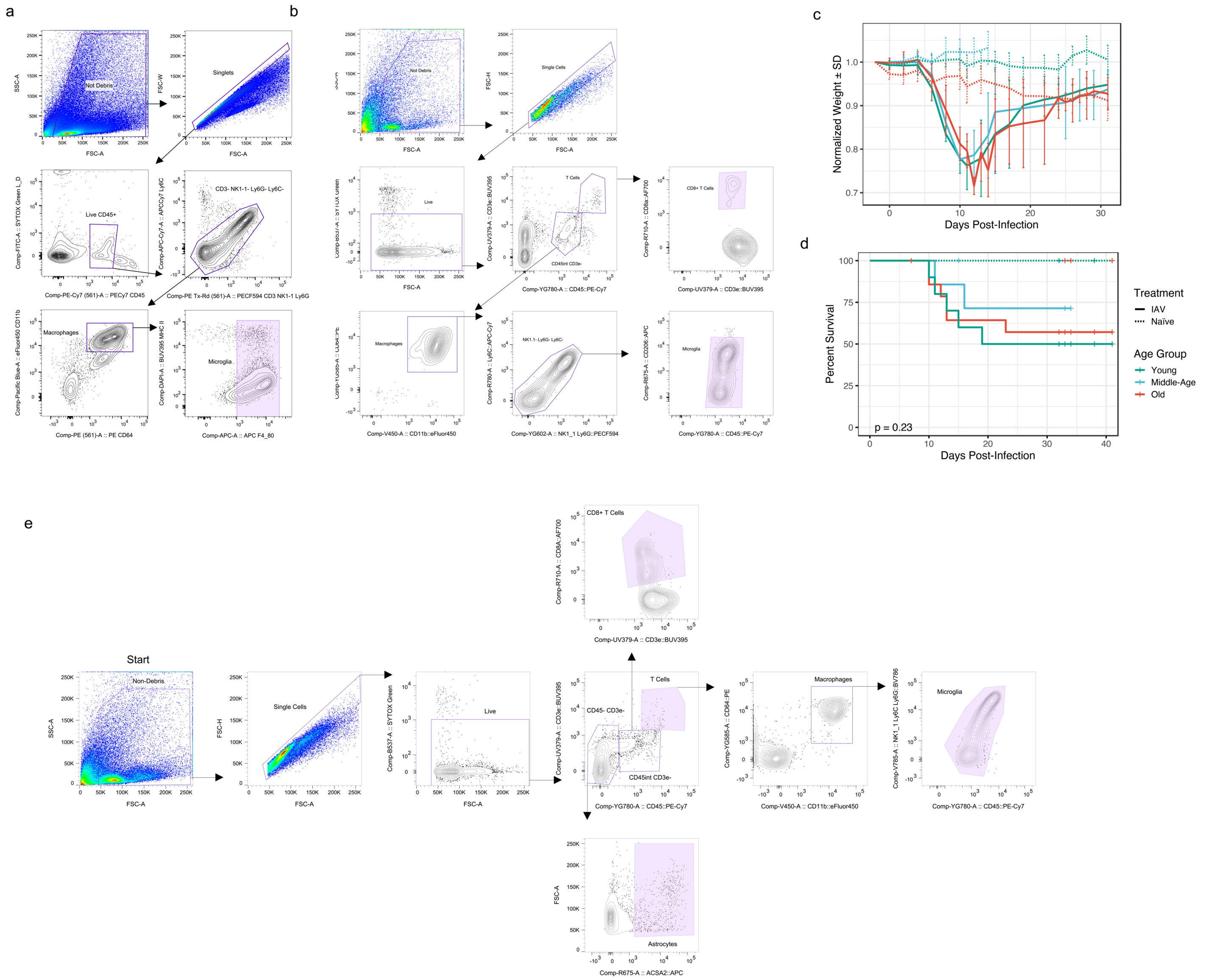

Figure S1. **Related to Figure 1. (a)** Representative gating for flow-cytometry sorting of microglia for bulk RNA-seq experiments in the absence of zsGreen expression. **(b)** Representative gating for flow-cytometry sorting of microglia for bulk metabolomics experiments. **(c)** Body weight measurements of C57BL/6JN mice in the steady state (naïve), during acute IAV pneumonia (1 weeks post-infection), and after recovery from pneumonia (5 weeks post-infection) in young adult (4-5mo; $n_{naïve}$ = 5, n_{acute} = 5, $n_{recovered}$ = 5), middle-aged (12-13mo; $n_{naïve}$ = 3, n_{acute} = 4, $n_{recovered}$ = 5), and old mice (18-19mo; $n_{naïve}$ = 4, n_{acute} = 8, $n_{recovered}$ = 8). Vertices are mean weights by group as a percent of starting weight with error bars indicating standard deviation. **(d)** Kaplan-Meier survival curves for all mice in (c). No significant difference in survival was observed between IAV-treated groups (p = 0.23, Kaplan-Meier). Plus signs (+) indicate mice censored due to harvest for neuroimmune cell isolation. **(e)** Representative gating for flow-cytometry sorting of neuroimmune cells for scRNA-seq experiments.

bioRxiv preprint doi: https://doi.org/10.64898/2025.12.11.693336; this version posted December 14, 2025. The copyright holder for this preprint (which was not certified by peer review) is the author/funder, who has granted bioRxiv a license to display the preprint in perpetuity. It is made available under aCC-BY 4.0 International license.

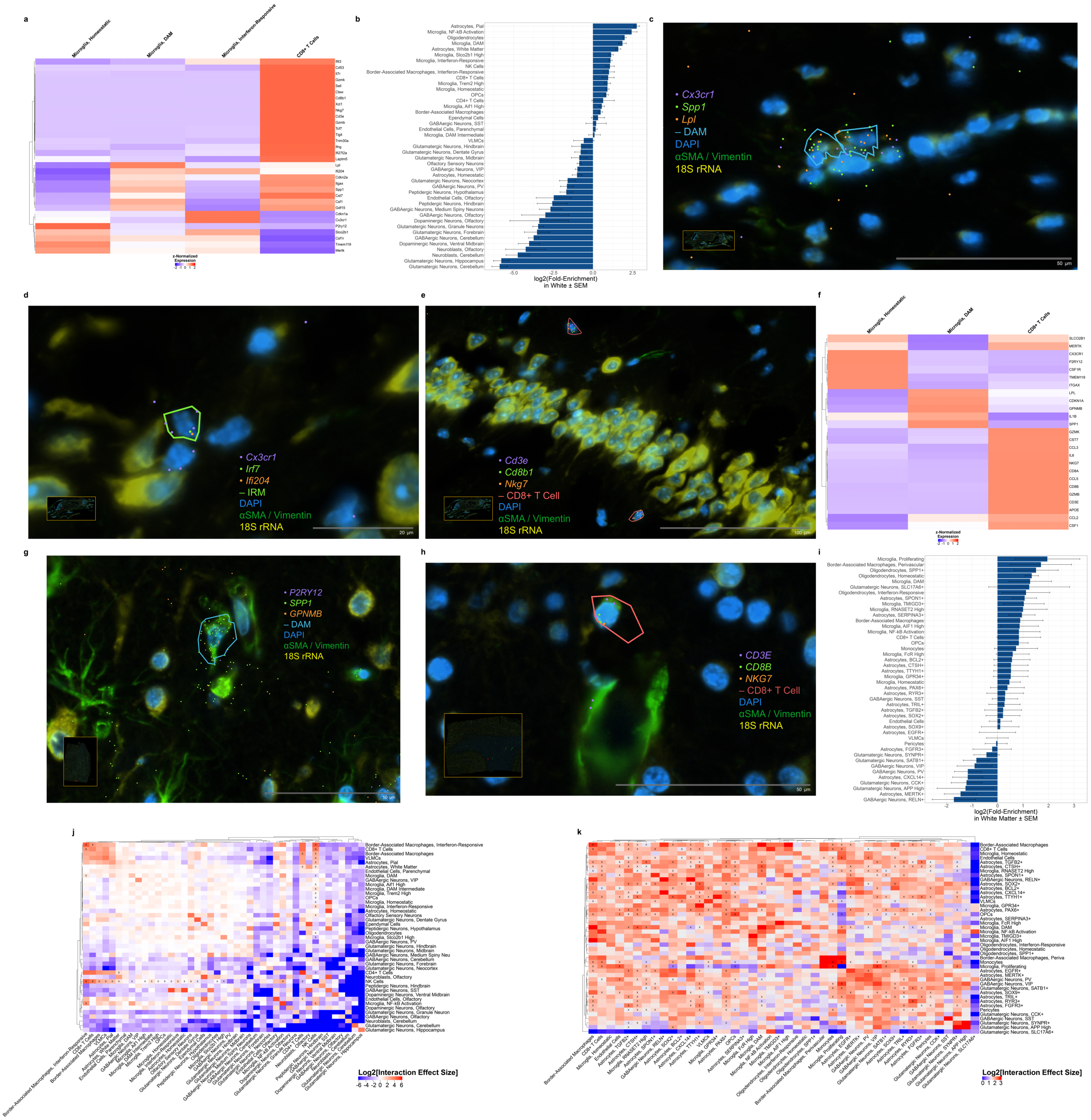

Figure S2. Related to Figure 2. (a) Hierarchical clustering of mean select marker gene expression by microglia and T cell state in imaging spatial transcriptomics data from (Fig. 2a). **(b)** Log2 fold-enrichment of all detected parenchymal cell states in white matter as compared to a random distribution for each mouse in (Fig. 2a). Only parenchymal cells were included in analysis. **(c)** Representative image of DAM in imaging spatial transcriptomics data. Purple dots: *Cx3cr1*; green dots: *Spp1*; orange dots: *Lpl*; cyan polygons: DAM identified by Baysor segmentation; blue: DNA (DAPI); green: αSMA/Vimentin; yellow: 18S rRNA. **(d)** Representative image of IRM in imaging spatial transcriptomics data. Purple dots: *Cx3cr1*; green dots: *Irf7*; orange dots: *Ifi204*; green polygons: IRM identified by Baysor segmentation; blue: DNA (DAPI); green: αSMA/Vimentin; yellow: 18S rRNA. **(e)** Representative image of CD8+ T cells invading the CA2

bioRxiv preprint doi: https://doi.org/10.64898/2025.12.11.693336; this version posted December 14, 2025. The copyright holder for this preprint (which was not certified by peer review) is the author/funder, who has granted bioRxiv a license to display the preprint in perpetuity. It is made available under aCC-BY 4.0 International license.

regions of the hippocampus in an old (18mo) animal. Purple dots: *CD3e*; green dots: *Cd8b1*; orange dots: *Nkg7*; red polygons: CD8+ T cells identified by Baysor segmentation; blue: DNA (DAPI); green: αSMA/Vimentin; yellow: 18S rRNA. **(f)** Hierarchical clustering of mean select marker gene expression by microglia and T cell state from human imaging spatial transcriptomics data in (Fig. 2f). **(g)** Representative image of DAM in human imaging spatial transcriptomics data. Purple dots: *P2RY12*; green dots: *SPP1*; orange dots: *GPNMB*; cyan polygons: DAM identified by Baysor segmentation; blue: DNA (DAPI); green: αSMA/Vimentin; yellow: 18S rRNA. **(h)** Representative image of CD8+ T in human imaging spatial transcriptomics data. Purple dots: *CD3E*; green dots: *CD8B*; orange dots: *NKG7*; red polygons: CD8+ T cells identified by Baysor segmentation; blue: DNA (DAPI); green: αSMA/Vimentin; yellow: 18S rRNA. **(i)** Log2 fold-enrichment of all detected cell states in white matter as compared to a random distribution for each patient in (Fig. 2f). **(j-k)** Spatial correlation between all cell states in whole mouse skulls (j) and postmortem human MFG (j). All correlations shown are significant ($q < 0.05$, global envelope test against a null hypothesis of random cell state distributions with FDR correction) except those marked with an “X” ($q \geq 0.05$).

bioRxiv preprint doi: https://doi.org/10.64898/2025.12.11.693336; this version posted December 14, 2025. The copyright holder for this preprint (which was not certified by peer review) is the author/funder, who has granted bioRxiv a license to display the preprint in perpetuity. It is made available under aCC-BY 4.0 International license.

a

IAV Gene	Total Counts in Dataset	Mean Counts per Cell	SD
HA	0	0	0
M1	0	0	0
M2	0	0	0
NA	0	0	0
NEP	0	0	0
NP	0	0	0
NS1	0	0	0
PA	0	0	0
PA-X	0	0	0
PB1	0	0	0
PB1-F2	0	0	0
PB2	0	0	0

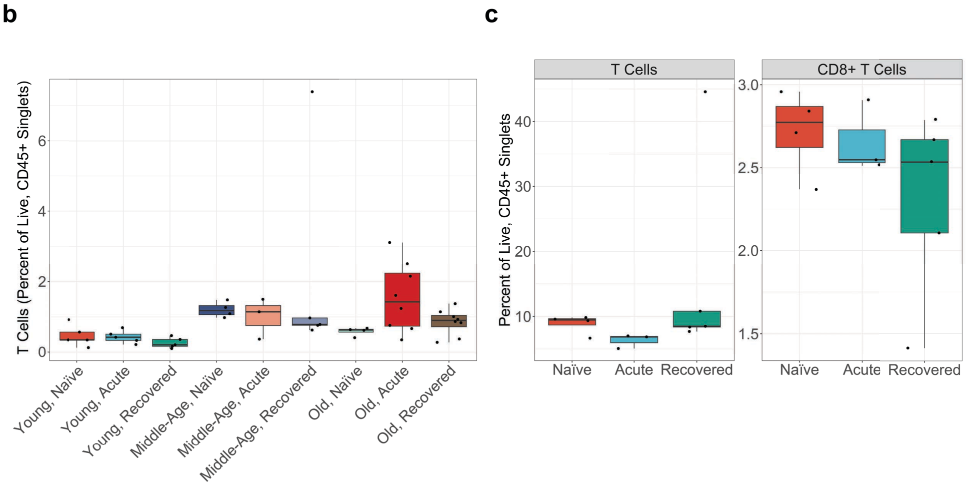

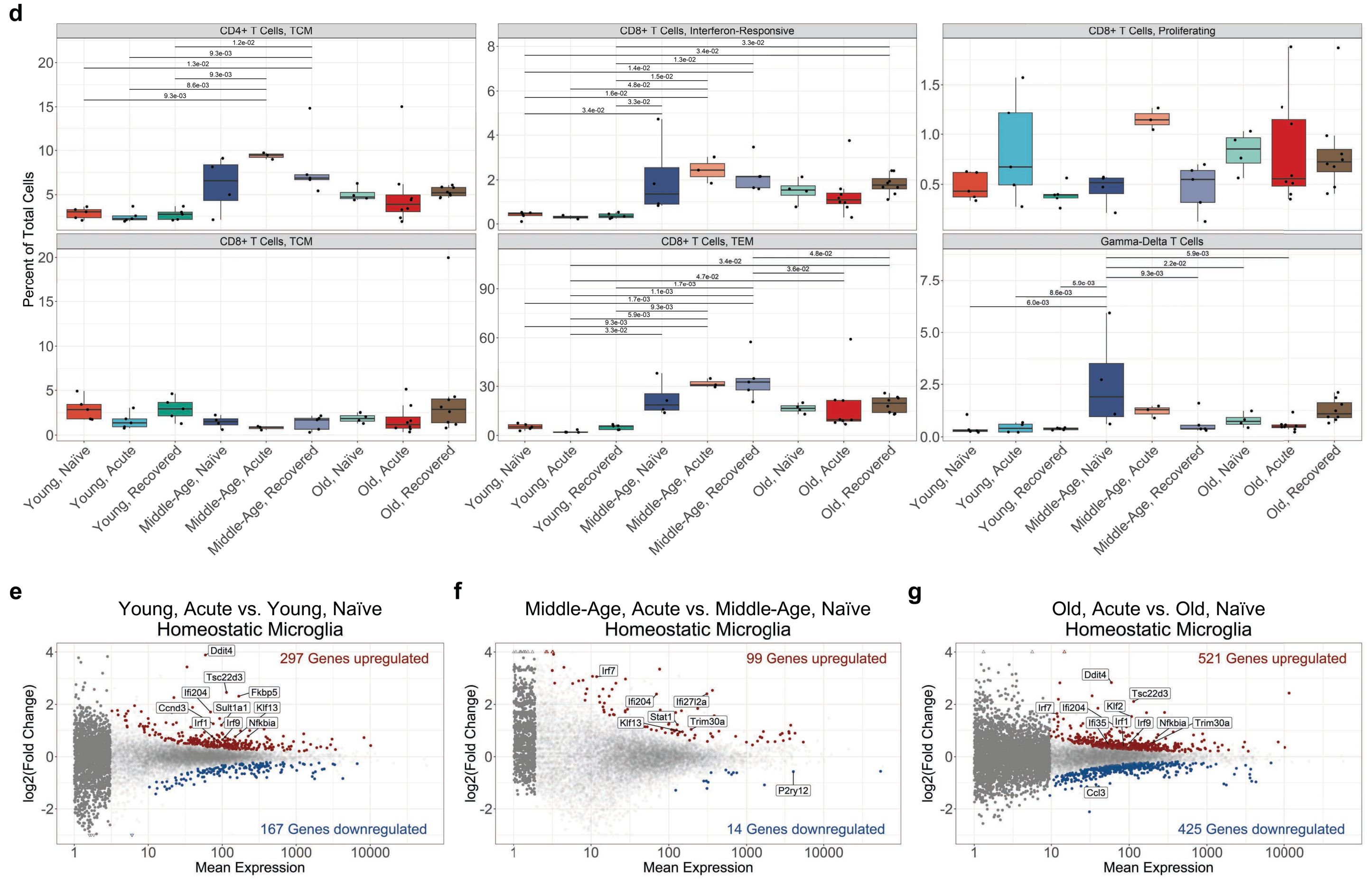

Figure S3. Related to Figure 3. (a) Table of summed IAV gene expression in all cells from scRNA-seq data. **(b)** Relative flow cytometric abundances of T cells from single-cell suspensions as a proportion of live, singlet, CD45$^+$ cells from whole mouse brains of C57BL/6JN mice in the steady state (naïve), during acute IAV infection (1 weeks post-infection), and after recovery from pneumonia (5 weeks post-infection) in young adult (4-5 months old; $n_{naïve} = 5$, $n_{acute} = 5$, $n_{recovered} = 5$), middle-aged (12-13 months old; $n_{naïve} = 3$, $n_{acute} = 4$, $n_{recovered} = 5$), and old age (18-19 months old; $n_{naïve} = 4$, $n_{acute} = 8$, $n_{recovered} = 8$). Comparisons are Wilcoxon rank sum tests with FDR correction. **(c)** Relative flow cytometric abundances of CD8+ T cells from single-cell suspensions as a proportion of live, singlet, CD45$^+$ cells from whole mouse brains of C57BL/6JN mice in the steady state (naïve), during acute IAV infection (1 weeks post-infection), and after recovery from pneumonia (5 weeks post-infection) in middle-aged (12-13 months old; $n_{naïve} = 3$, $n_{acute} = 4$, $n_{recovered} = 5$) mice. Comparisons are

bioRxiv preprint doi: https://doi.org/10.64898/2025.12.11.693336; this version posted December 14, 2025. The copyright holder for this preprint (which was not certified by peer review) is the author/funder, who has granted bioRxiv a license to display the preprint in perpetuity. It is made available under aCC-BY 4.0 International license.

Wilcoxon rank sum tests with FDR correction. **(d)** Relative abundances of selected T cell states as a percentage of total cells in scRNA-seq dataset. Comparisons are t-tests with FDR correction. **(e-g)** MA plots of pseudo-bulk RNA-seq data derived from scRNA-seq data comparing only homeostatic microglia transcriptomic responses to IAV pneumonia in (d) young adult, (e) middle-aged, and (f) old mice. Significantly ($q < 0.05$, Wald test) upregulated genes are shown in red, and significantly downregulated genes are shown in blue. Genes shown in gray are not significantly differentially expressed. Genes represented by triangles are outside the plot limits.

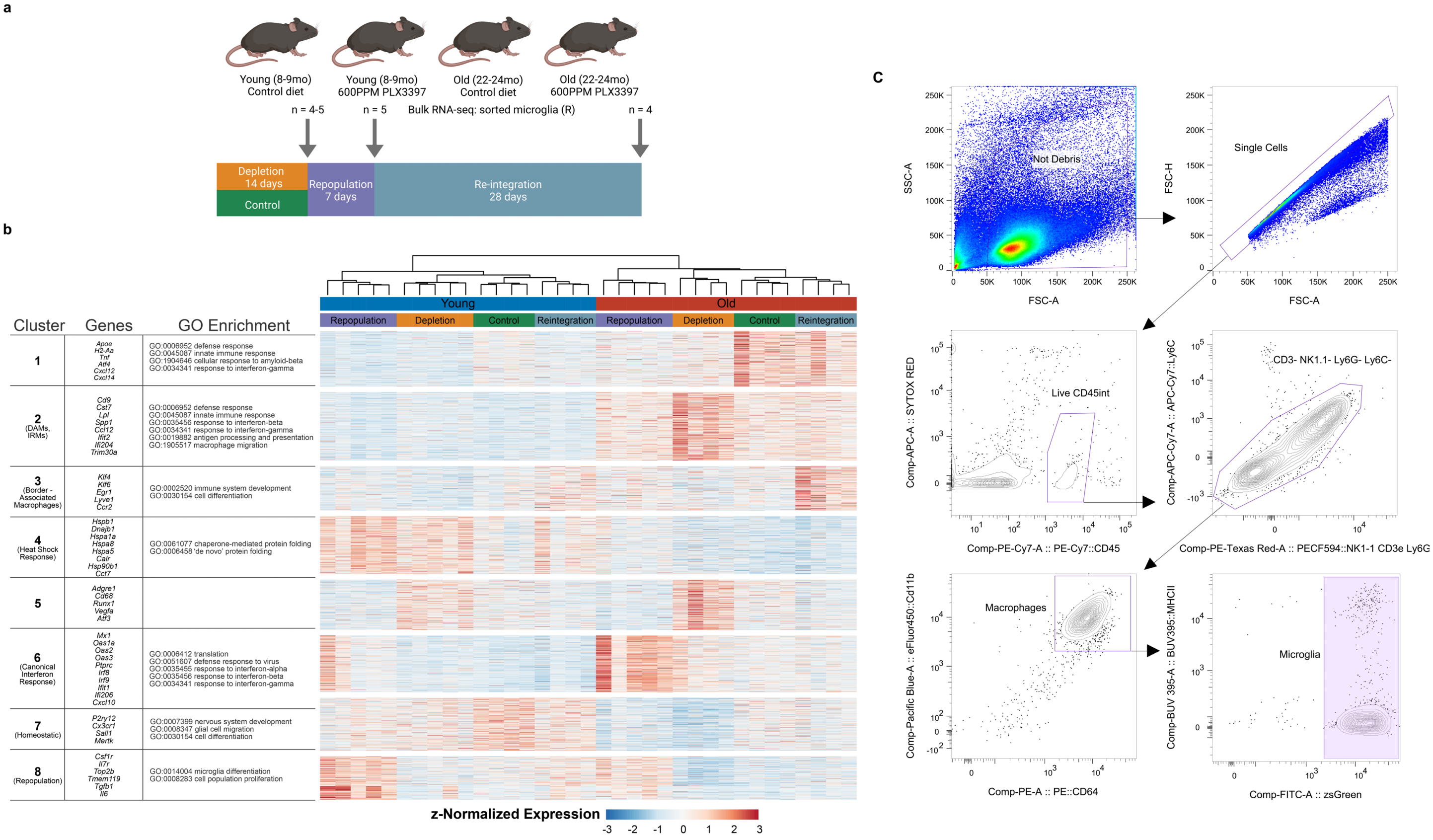

Figure S4. Related to Figure 4. (a) Visual methods for microglial depletion and repopulation experiments for control chow: young adult (8mo; n = 4), old (23mo; n = 4); 14-day depletion with 600ppm PLX3397 chow: young adult (n = 4; 8mo), old (n = 4; 22mo), 7 days after PLX3397 withdrawal: young adult (n = 5; 8mo), old (n = 5; 21mo), and 30 days after PLX3397 withdrawal: young adult (n = 4; 8mo), old (n = 4; 22mo). **(b)** Hierarchical clustering of z-normalized gene expression for all mice in Figure 4a-c. All significantly variable genes ($q < 0.05$, likelihood ratio test) are shown. Samples (columns) were clustered using Ward's method (D2) and genes (rows) were clustered using k-means clustering. Select genes and significantly enriched gene ontology (GO) annotations ($q < 0.05$, Fisher's exact test with FDR correction) are shown at left. **(c)** Representative gating for flow cytometry sorting of microglia for bulk RNA-seq experiments in Figure 4d-e.

Supplementary Table S1. List of reagents used for flow cytometry sorting of mouse microglia without fluorescent reporters for bulk RNA-seq.

Laser	Filter	Dye	Antigen	Clone	Dilution	Catalog Number	RR ID

bioRxiv preprint doi: https://doi.org/10.64898/2025.12.11.693336; this version posted December 14, 2025. The copyright holder for this preprint (which was not certified by peer review) is the author/funder, who has granted bioRxiv a license to display the preprint in perpetuity. It is made available under aCC-BY 4.0 International license.

305	379/28	BUV395	MHC II	2G9	1:500	BD 743876	AB_2741827
488	530/30	SYTOX Green			1:1000	Invitrogen S34860	
405	450/50	eFluor450	CD11b	M1/70	1:400	Invitrogen 48-0112-82	AB_1582236
640	670/30	APC	F4/80	BM8	1:500	Invitrogen 17-4801-82	AB_2784648
640	780/60	APC-Cy7	Ly6C	HK1.4	1:1000	BioLegend 128026	AB_10640120
552	575/25	PE	CD64	X54-5/7.1	1:1000	BioLegend 139304	AB_10612740
552	610/20	PE-CF594	NK1.1	PK136	1:2000	BD 562864	AB_2737850
552	610/20	PE-CF594	CD3e	145-2C11	1:333.33	BD 562286	AB_11153307
552	610/20	PE-CF594	Ly6G	1A8	1:2000	BD 562700	AB_2737730
552	780/60	PECy7	CD45	30-F11	1:1000	BD 552848	AB_394489

Supplementary Table S2. List of reagents used for flow cytometry sorting of mouse microglia without fluorescent reporters for bulk metabolomics.

Laser	Filter	Dye	Antigen	Clone	Dilution	Catalog Number	RR ID
305	379/28	BUV395	CD3e	145-2C11	1:100	BD 563565	AB_2738278
488	530/30	SYTOX Green			1:1000	Invitrogen S34860	
405	450/50	eFluor450	CD11b	M1/70	1:400	Invitrogen 48-0112-82	AB_1582236

bioRxiv preprint doi: https://doi.org/10.64898/2025.12.11.693336; this version posted December 14, 2025. The copyright holder for this preprint (which was not certified by peer review) is the author/funder, who has granted bioRxiv a license to display the preprint in perpetuity. It is made available under aCC-BY 4.0 International license.

640	670/30	APC	CD206	C068C2	1:500	BioLegend 141708	AB_10900231
640	780/60	APC-Cy7	Ly6C	HK1.4	1:1000	BioLegend 128026	AB_10640120
640	670/30	AF700	CD8a	53-6.7	1:333	BD 557959	AB_396959
552	575/25	PE	CD64	X54-5/7.1	1:1000	BioLegend 139304	AB_10612740
552	610/20	PE-CF594	NK1.1	PK136	1:2000	BD 562864	AB_2737850
552	610/20	PE-CF594	Ly6G	1A8	1:2000	BD 562700	AB_2737730
552	780/60	PECy7	CD45	30-F11	1:1000	BD 552848	AB_394489

Supplementary Table S3. List of reagents used for flow cytometry sorting of mouse neuro-immune cells for scRNA-seq.

Laser	Filter	Dye	Antigen	Clone	Dilution	Catalog Number	RR ID
305	379/28	BUV395	CD3e	145-2C11	1:100	BD 563565	AB_2738278
488	530/30	FITC	SYTOX Green		1:1000	Invitrogen S34860	
405	780/60	BV786	NK1.1	PK136	1:333.33	BD 740853	AB_2740506
405	780/60	BV785	Ly6G	1A8	1:100	BioLegend 127645	AB_2566317
405	780/60	BV785	Ly6C	HK1.4	1:1000	BioLegend 128041	AB_2565852
405	450/50	eFluor450	CD11b	M1/70	1:400	Invitrogen 48-0112-82	AB_1582236
640	670/30	APC	ACSA-2	IH3-18A3	1:200	130–117-386	AB_2727930

bioRxiv preprint doi: https://doi.org/10.64898/2025.12.11.693336; this version posted December 14, 2025. The copyright holder for this preprint (which was not certified by peer review) is the author/funder, who has granted bioRxiv a license to display the preprint in perpetuity. It is made available under aCC-BY 4.0 International license.

552	575/25	PE	CD64	X54-5/7.1	1:1000	BioLegend 139304	AB_10612 740
552	780/60	PECy7	CD45	30-F11	1:1000	BD 552848	AB_39448 9

Supplementary Table S4. Cohort demographics for human MFG spatial transcriptomics analysis.

Supplementary Table 4: Human brain aging cohort demographics

Case Number	Clinical Diagnosis	Primary Neuropathologic Diagnoses	NIA–AA ADNC (ABC Score)	ApoE	PMI (Hrs)	Sex	Age at Death	Race/Ethnicity	Group
1	Amnestic dementia	Intermediate ADNC, LATE stage 2	A3, B2, C2	3,3	8.5	Female	95	Caucasian/non-hispanic	ADNC + LATE-NC, Old
2	SuperAger	Intermediate ADNC, LATE stage 2	A3, B2, C3	3,3	20	Female	97	Caucasian/non-hispanic	ADNC + LATE-NC, Old
3	SuperAger	Low ADNC	A1, B1, C1	3,3	6	Male	91	Caucasian/non-hispanic	SuperAger, Old
4	Amnestic dementia	High ADNC, LATE stage 2	A3, B3, C3	3,4	11	Female	75	Caucasian/non-hispanic	ADNC + LATE-NC, Old
5	SuperAger	PART, LATE stage 2	A0, B2, C0	3,3	16	Male	87	Caucasian/non-hispanic	SuperAger, Old
6	SuperAger	PART	A0, B2, C0	3,3	9	Female	85	Caucasian/non-hispanic	SuperAger, Old
7	Young control	None	None	Unavailable	Unavailable	Male	28	Unavailable	Normal Control, Young Adult
8	Normal control	Intermediate ADNC, LATE stage 1	A3, B2, C1	3,4	21	Male	92	Caucasian/non-hispanic	Normal Control, Old
9	Amnestic dementia	High ADNC	A3, B3, C3	3,3	16	Male	62	Caucasian/non-hispanic	ADNC, Old
10	Amnestic dementia	High ADNC	A3, B3, C3	Unavailable	18	Male	66	Caucasian/non-hispanic	ADNC, Old
11	Normal control	Low ADNC	A3, B1, C3	3,3	16	Female	87	Caucasian/non-hispanic	Normal Control, Old

Supplementary Table S5. List of reagents used for flow cytometry sorting of mouse microglia expressing zsGreen for bulk RNA-seq.

Laser	Filter	Dye	Antigen	Clone	Dilution	Catalog Number	RR ID
305	379/28	BUV395	MHC II	2G9	1:500	BD 743876	AB_27418 27
488	530/30	zsGreen					
405	450/50	eFluor450	CD11b	M1/70	1:400	Invitrogen 48-0112-82	AB_15822 36
640	670/30	SYTOX Red			1:1000	Invitrogen S34859	
640	780/60	APC-Cy7	Ly6C	HK1.4	1:1000	BioLegend 128026	AB_10640 120
552	575/25	PE	CD64	X54-5/7.1	1:1000	BioLegend 139304	AB_10612 740

bioRxiv preprint doi: https://doi.org/10.64898/2025.12.11.693336; this version posted December 14, 2025. The copyright holder for this preprint (which was not certified by peer review) is the author/funder, who has granted bioRxiv a license to display the preprint in perpetuity. It is made available under aCC-BY 4.0 International license.

552	610/20	PE-CF594	NK1.1	PK136	1:2000	BD 562864	AB_2737850
552	610/20	PE-CF594	CD3e	145-2C11	1:333.33	BD 562286	AB_11153307
552	610/20	PE-CF594	Ly6G	1A8	1:2000	BD 562700	AB_2737730
552	780/60	PECy7	CD45	30-F11	1:1000	BD 552848	AB_394489

959
