# Supplementary material for: Influenza A infection accelerates disease-associated microglia formation during physiological aging
Source: bioRxiv. 2025 Dec 14:2025.12.11.693336. Preprint. [Version 1] doi: 10.64898/2025.12.11.693336 (PMC12710644; doi:10.64898/2025.12.11.693336)
Supplement: Supplement 6 [file media-6.pdf]

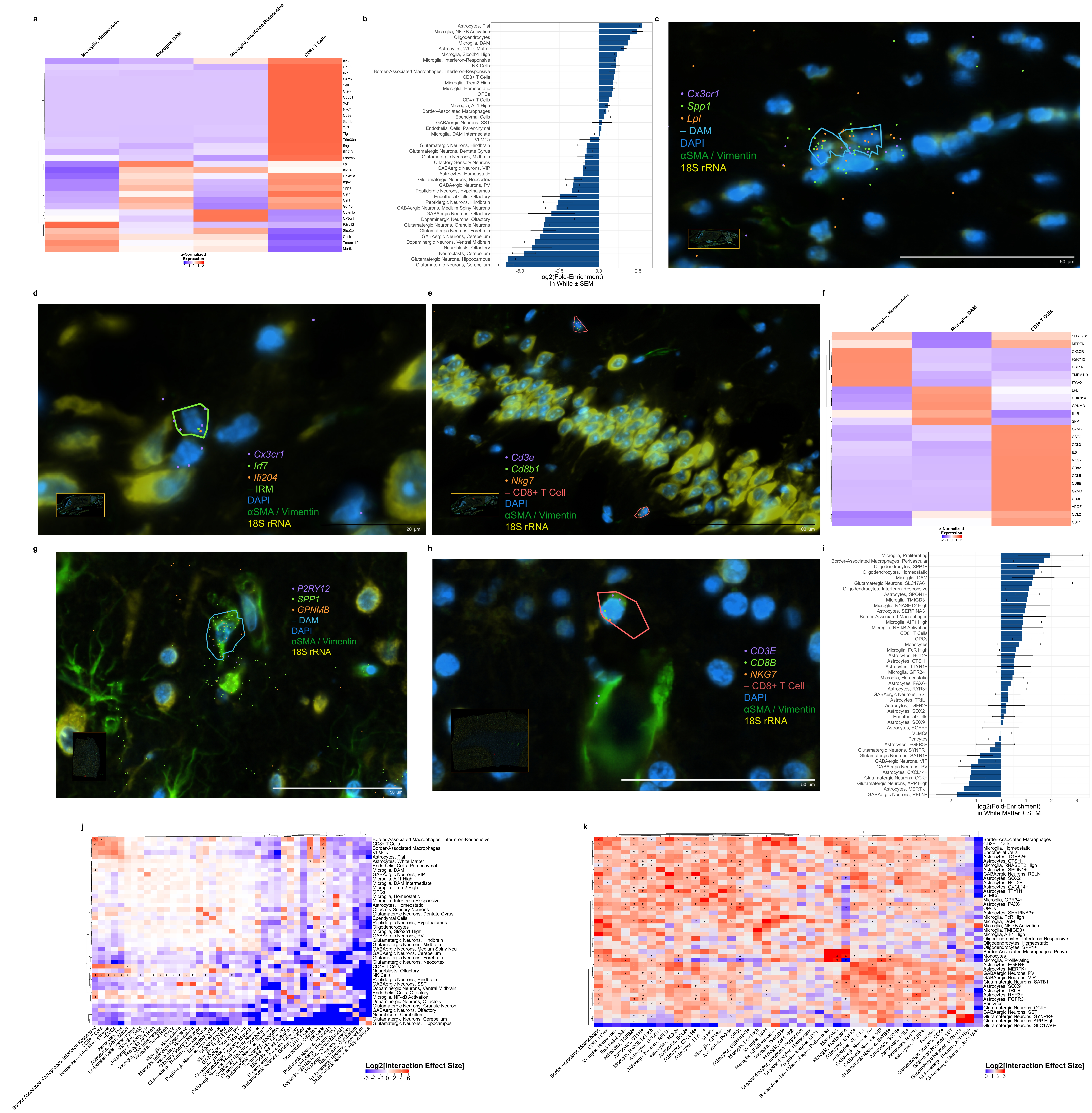
a
Microglia, Homeostatic
Microglia, DAM
Microglia, Interferon-Responsive
CD8+ T Cells
Ifit3
Cd53
Il7r
Gzmk
Sell
Ctsw
Cd8b1
Xcl1
Nkg7
Cd3e
Gzmb
Tcf7
Tigit
Trim30a
Ifng
Ifi27l2a
Laptm5
Lpl
Ifi204
Cdkn2a
Itgax
Spp1
Cst7
Csf1
Gdf15
Cdkn1a
Cx3cr1
P2ry12
Slco2b1
Csf1r
Tmem119
Mertk
z-Normalized Expression
-2 -1 0 1 2
b
Astrocytes, Pial
Microglia, NF-kB Activation
Oligodendrocytes
Microglia, DAM
Astrocytes, White Matter
Microglia, Slco2b1 High
Microglia, Interferon-Responsive
NK Cells
Border-Associated Macrophages, Interferon-Responsive
CD8+ T Cells
Microglia, Trem2 High
Microglia, Homeostatic
OPCs
CD4+ T Cells
Microglia, Aif1 High
Border-Associated Macrophages
Ependymal Cells
GABAergic Neurons, SST
Endothelial Cells, Parenchymal
Microglia, DAM Intermediate
VLMCs
Glutamatergic Neurons, Hindbrain
Glutamatergic Neurons, Dentate Gyrus
Glutamatergic Neurons, Midbrain
Olfactory Sensory Neurons
GABAergic Neurons, VIP
Astrocytes, Homeostatic
Glutamatergic Neurons, Neocortex
GABAergic Neurons, PV
Peptidergic Neurons, Hypothalamus
Endothelial Cells, Olfactory
Peptidergic Neurons, Hindbrain
GABAergic Neurons, Medium Spiny Neurons
GABAergic Neurons, Olfactory
Dopaminergic Neurons, Olfactory
Glutamatergic Neurons, Granule Neurons
Glutamatergic Neurons, Forebrain
GABAergic Neurons, Cerebellum
Dopaminergic Neurons, Ventral Midbrain
Neuroblasts, Olfactory
Neuroblasts, Cerebellum
Glutamatergic Neurons, Hippocampus
Glutamatergic Neurons, Cerebellum
-5.0 -2.5 0.0 2.5
log2(Fold-Enrichment) in White ± SEM
c
• Cx3cr1
• Spp1
• Lpl
– DAM
DAPI
αSMA / Vimentin
18S rRNA
50 μm
d
• Cx3cr1
• Irf7
• Ifi204
– IRM
DAPI
αSMA / Vimentin
18S rRNA
20 μm
e
• Cd3e
• Cd8b1
• Nkg7
– CD8+ T Cell
DAPI
αSMA / Vimentin
18S rRNA
100 μm
f
Microglia, Homeostatic
Microglia, DAM
CD8+ T Cells
SLCO2B1
MERTK
CX3CR1
P2RY12
CSF1R
TMEM119
ITGAX
LPL
CDKN1A
GPNMB
IL1B
SPP1
GZMK
CST7
CCL3
IL6
NKG7
CD8A
CCL5
CD8B
GZMB
CD3E
APOE
CCL2
CSF1
z-Normalized Expression
-2 -1 0 1 2
g
• P2RY12
• SPP1
• GPNMB
– DAM
DAPI
αSMA / Vimentin
18S rRNA
50 μm
h
• CD3E
• CD8B
• NKG7
– CD8+ T Cell
DAPI
αSMA / Vimentin
18S rRNA
50 μm
i
Microglia, Proliferating
Border-Associated Macrophages, Perivascular
Oligodendrocytes, SPP1+
Oligodendrocytes, Homeostatic
Microglia, DAM
Glutamatergic Neurons, SLC17A6+
Oligodendrocytes, Interferon-Responsive
Astrocytes, SPON1+
Microglia, TMIGD3+
Microglia, RNASET2 High
Astrocytes, SERPINA3+
Border-Associated Macrophages
Microglia, AIF1 High
Microglia, NF-kB Activation
CD8+ T Cells
OPCs
Monocytes
Microglia, FcR High
Astrocytes, BCL2+
Astrocytes, CTSH+
Astrocytes, TTYH1+
Microglia, GPR34+
Microglia, Homeostatic
Astrocytes, PAX6+
Astrocytes, RYR3+
GABAergic Neurons, SST
Astrocytes, TRIL+
Astrocytes, TGFB2+
Astrocytes, SOX2+
Endothelial Cells
Astrocytes, SOX9+
Astrocytes, EGFR+
VLMCs
Pericytes
Astrocytes, FGFR3+
Glutamatergic Neurons, SYNPR+
Glutamatergic Neurons, SATB1+
GABAergic Neurons, VIP
GABAergic Neurons, PV
Astrocytes, CXCL14+
Glutamatergic Neurons, CCK+
Glutamatergic Neurons, APP High
Astrocytes, MERTK+
GABAergic Neurons, RELN+
-2 -1 0 1 2 3
log2(Fold-Enrichment) in White Matter ± SEM
j
Border-Associated Macrophages, Interferon-Responsive
CD8+ T Cells
Border-Associated Macrophages
VLMCs
Astrocytes, Pial
Astrocytes, White Matter
Endothelial Cells, Parenchymal
Microglia, DAM
GABAergic Neurons, VIP
Microglia, Aif1 High
Microglia, DAM Intermediate
Microglia, Trem2 High
OPCs
Microglia, Homeostatic
Microglia, Interferon-Responsive
Astrocytes, Homeostatic
Olfactory Sensory Neurons
Glutamatergic Neurons, Dentate Gyrus
Ependymal Cells
Peptidergic Neurons, Hypothalamus
Oligodendrocytes
Microglia, Slco2b1 High
GABAergic Neurons, PV
Glutamatergic Neurons, Hindbrain
Glutamatergic Neurons, Midbrain
GABAergic Neurons, Medium Spiny Neu
GABAergic Neurons, Cerebellum
Glutamatergic Neurons, Forebrain
Glutamatergic Neurons, Neocortex
CD4+ T Cells
Neuroblasts, Olfactory
NK Cells
Peptidergic Neurons, Hindbrain
GABAergic Neurons, SST
Dopaminergic Neurons, Ventral Midbrain
Endothelial Cells, Olfactory
Microglia, NF-kB Activation
Dopaminergic Neurons, Olfactory
Glutamatergic Neurons, Granule Neuron
GABAergic Neurons, Olfactory
Neuroblasts, Cerebellum
Glutamatergic Neurons, Cerebellum
Glutamatergic Neurons, Hippocampus
Log2[Interaction Effect Size]
-6 -4 -2 0 2 4 6
k
Border-Associated Macrophages
CD8+ T Cells
Microglia, Homeostatic
Endothelial Cells
Astrocytes, TGFB2+
Astrocytes, CTSH+
Microglia, RNASET2 High
Astrocytes, SPON1+
GABAergic Neurons, RELN+
Astrocytes, SOX2+
Astrocytes, BCL2+
Astrocytes, CXCL14+
Astrocytes, TTYH1+
VLMCs
Microglia, GPR34+
Astrocytes, PAX6+
OPCs
Astrocytes, SERPINA3+
Microglia, FcR High
Microglia, DAM
Microglia, NF-kB Activation
Microglia, TMIGD3+
Microglia, AIF1 High
Oligodendrocytes, Interferon-Responsive
Oligodendrocytes, Homeostatic
Oligodendrocytes, SPP1+
Border-Associated Macrophages, Periva
Monocytes
Microglia, Proliferating
Astrocytes, EGFR+
Astrocytes, MERTK+
GABAergic Neurons, PV
GABAergic Neurons, VIP
Glutamatergic Neurons, SATB1+
Astrocytes, SOX9+
Astrocytes, TRIL+
Astrocytes, RYR3+
Astrocytes, FGFR3+
Pericytes
Glutamatergic Neurons, CCK+
GABAergic Neurons, SST
Glutamatergic Neurons, SYNPR+
Glutamatergic Neurons, APP High
Glutamatergic Neurons, SLC17A6+
Log2[Interaction Effect Size]
0 1 2 3
